# Acod1 Promotes PAD4 Ubiquitination via UBR5 Alkylation to Modulate NETosis and Exert Protective Effects in Sepsis

**DOI:** 10.1002/advs.202411652

**Published:** 2025-06-30

**Authors:** Huifan Liu, Guoqing Jing, Shujuan Wu, Min Yuan, Yingyue Dong, Xue Chen, Huimin Zhou, Hailong Gong, Jing Zuo, Xiaojing Wu, Xuemin Song

**Affiliations:** ^1^ Department of Anesthesiology, Research Centre of Anesthesiology and Critical Care Medicine Zhongnan Hospital of Wuhan University Wuhan Hubei 430071 China; ^2^ Department of Anesthesiology Renmin Hospital of Wuhan University Wuhan Hubei 430060 China; ^3^ Department of Respiratory and Critical Care Medicine Renmin Hospital of Wuhan University Wuhan 430060 China

**Keywords:** Acod1, Neutrophil extracellular traps, Neutrophils, Sepsis, Ubiquitination

## Abstract

Neutrophil extracellular traps (NETs) are reticular structures released by neutrophils, and the process of their formation is called NETosis. NETs play a key role in the pathological process of sepsis. However, the specific regulatory mechanism has not been fully clarified. This study finds that the levels of NETs in peripheral blood are significantly elevated in clinical sepsis patients and cecal ligation and puncture (CLP) mouse models, and the expression of Acod1 is closely related to the generation of NETs. Acod1 knockout led to a further increase in NETs levels in CLP mice, aggravated the inflammatory response, worsened organ damage, and reduced the survival rate. Further studies indicate that E3 ubiquitin ligase UBR5 interacts with PAD4 (one of the core proteins for NETs generation). Acod1/itaconate (ITA) enhanced the enzymatic activity of UBR5 through alkylation modification, promoting the K48‐linked polyubiquitination and degradation of PAD4, thereby inhibiting NETosis. In conclusion, this study combines transcriptomics, metabolomics, genetic engineering, and co‐immunoprecipitation techniques to reveal the molecular mechanism of Acod1/ITA in regulating NETs, providing new potential targets and theoretical basis for the treatment of sepsis.

## Graphical abstract

In this study, Acod1 knockout in CLP mice significantly increases peripheral blood NET levels, exacerbating inflammation, organ damage, and reducing survival. Further research shows that UBR5 interacts with PAD4, a key NET formation protein. Acod1/itaconate (ITA) enhances the enzymatic activity of UBR5 by alkylating the Cys2768 site, promoting the K48‐linked polyubiquitination and degradation of PAD4, thereby inhibiting NETosis.

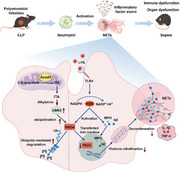

## 1 Introduction

Sepsis is a condition where infection leads to a systemic immune dysfunction, resulting in multiple organ failure and even death.^[^ ^1^ ^]^ It is reported that there are over 48.9 million new cases of sepsis globally each year, with sepsis‐related deaths reaching 11 million, accounting for 19.7% of total global deaths.^[^ ^2^ ^]^ In recent years, despite the increasing research on sepsis, the morbidity and mortality rates among sepsis patients remain high.^[^ ^3^ ^]^ Therefore, further exploration of the pathogenic mechanisms of sepsis and the search for more effective prevention and treatment strategies have become critical scientific issues in this field.

Neutrophils are the most abundant innate immune cells in the human body.^[^ ^4^ ^]^ Neutrophil extracellular traps (NETs) are web‐like structures produced by neutrophils, composed of a DNA backbone along with attached components such as histones, myeloperoxidase (MPO), and neutrophil elastase (NE).^[^ ^5^ ^]^ The process by which activated neutrophils release NETs is termed NETosis.^[^ ^6^ ^]^ NETs are crucial for host defense because they can trap and kill extracellular pathogens.^[^ ^7^ ^]^ However, during sepsis, large amounts of NETs in the peripheral circulation are degraded by DNases, releasing large quantities of damage‐associated molecular patterns (DAMPs) such as histones, cell‐free DNA (cfDNA), and MPO, leading to tissue and vascular damage and exacerbating the progression of sepsis.^[^ ^8^ ^]^ The initiation of NETosis involves the activation of peptidylarginine deiminase 4 (PAD4).^[^ ^9^ ^]^ The citrullination of histones mediated by PAD4 is the core mechanism of NETosis.^[^ ^10^ ^]^ Under stimulation by lipopolysaccharide (LPS), bacteria, or calcium ionophores, Ca^2+^ influx into neutrophils triggers NADPH phosphorylation, generating large amounts of reactive oxygen species (ROS).^[^ ^11^ ^]^ ROS lead to the activation and translocation of PAD4 into the nucleus, further causing the transfer of NE and MPO from azurophilic granules to the nucleus.^[^ ^12^ ^]^ Together, MPO, NE, and PAD4 contribute to histone citrullination and chromatin decondensation, ultimately leading to the formation of NETs.^[^ ^13^ ^]^ Although PAD4 plays a vital role in the production of NETs by neutrophils, the regulatory mechanisms underlying this process remain to be fully elucidated.

Aconitate decarboxylase (Acod1) catalyzes the decarboxylation of aconitate in the mitochondrial matrix, producing itaconate (ITA).^[^ ^14^ ^]^ Under normal conditions, the expression levels of Acod1 within the cell are low, resulting in relatively low concentrations of ITA.^[^ ^15^ ^]^ However, when the body is subjected to external stimuli such as oxidative stress or infection, myeloid immune cells undergo metabolic reprogramming, reducing the conversion of ITA into acetyl‐CoA and leading to ITA accumulation.^[^ ^16^ ^]^ ITA transfers from the mitochondrial matrix to the cytoplasm, playing various regulatory roles in immune responses.^[^ ^17^ ^]^ Alkylation refers to the transfer of alkyl groups, such as methyl or ethyl, to specific residues of a protein, altering its function.^[^ ^18^ ^]^ Studies had shown that one key mechanism by which Acod1/ITA regulates the immune response is through the alkylation of target proteins.^[^ ^19^ ^]^ ITA, being an electrophilic α, β‐unsaturated carboxylic acid, can alkylate the cysteine residues of proteins via Michael addition reactions, forming 2, 3‐dicarbonyl propyl adducts, thereby altering protein function.^[^ ^20^ ^]^ Research had demonstrated that the Acod1‐ITA axis can downregulate hypoxia‐inducible factor 1α (HIF‐1α) in neutrophils and upregulate heme oxygenase, exerting certain anti‐inflammatory effects.^[^ ^21^ ^]^ However, the precise role of the Acod1/ITA axis in sepsis and NETosis remains unclear.

In this study, we first revealed the close association between Acod1 and neutrophil NET formation in sepsis. We also elucidated a novel mechanism wherein Acod1/ITA enhanced the enzymatic activity of the E3 ubiquitin ligase UBR5 through alkylation modification, thereby promoting K48‐linked polyubiquitination and degradation of PAD4, which in turn inhibited NET formation. Furthermore, in a cecal ligation and puncture (CLP) mouse model, we demonstrated that targeting Acod1 in neutrophils effectively treated sepsis. This new therapeutic approach held promise as a key strategy in the clinical management of sepsis.

## 2 Experimental Section

### 2.1 Ethics Statement

The study protocol was approved by the Medical Ethics Committee of Renmin Hospital of Wuhan University (WDRY2022‐K046). All enrolled participants signed the written informed consent form before being included in the study. The baseline data of the patients are shown in Table S1 (Supporting Information). All animal experiments conducted adhered strictly to National Institutes of Health (NIH) standards and received approval from Wuhan University's Animal Ethics Committee (Ethical Approval Number ZN2023186).

### 2.2 Recruitment of Healthy Volunteers and Clinical Sepsis Patients

Patients diagnosed as sepsis according to the sepsis 3.0 criteria were included in this study. From March 2023 to March 2024, blood samples were collected from 20 sepsis patients and 20 healthy volunteers in the Intensive Care Unit of Renmin Hospital of Wuhan University.

### 2.3 Mice and Treatment

This study involved male C57BL/6 J wild‐type mice (WT) and Acod1 gene knockout mice (Acod1^‐/‐^), both 7‐8 weeks old and weighing between 22 and 25 grams. The mice were maintained in a 12‐h light/dark cycle environment with temperatures ranging from 22 to 27°C and humidity levels between 45% and 55%, with unrestricted access to food and water. To thoroughly validate our hypothesis, we established sepsis mouse models using two different methods: intraperitoneal LPS injection and cecal ligation and puncture (CLP). The LPS‐induced sepsis model was established by administering 10 mg kg^−1^ LPS intraperitoneally (i.p.) or 5 mg kg^−1^ LPS via intratracheal instillation, with assessment performed within 24 h post‐exposure. Control mice received an equal volume of sterile saline either intraperitoneally or via intratracheal instillation. The CLP sepsis model was briefly established as follows: an abdominal skin incision was made under sterile conditions to expose the cecum. The cecum was then ligated 75% with a 6‐0 silk suture and punctured with a 21‐gauge needle. For sham‐operated mice, all procedures were identical except that the cecum was not ligated or punctured. Post‐surgery, mice received 1 mL of saline intraperitoneally to restore fluid balance.

### 2.4 Materials

Lipopolysaccharide (LPS) were purchased from MedChemExpress (Monmouth Junction, NJ, USA). For in vitro cell culture, fetal bovine serum (FBS), RPMI 1640, DMEM, DMEM/F12 medium, PBS, trypsin‐EDTA, and penicillin/streptomycin (P/S) were purchased from Cytiva Bio‐technology Co., Ltd (Washington, USA). The HL60 cell line was provided by Wuhan Pricella Biotechnology Co., Ltd (Wuhan, China). Triton X‐100 and DAPI for cell staining were sourced from Sigma‒Aldrich. The radioimmunoprecipitation lysis buffer (RIPA), and bicinchoninic acid (BCA) protein detection kit were purchased from Jiangsu Beitian Biotechnology Co., Ltd. The antibodies used in the study are shown in Table S2 (Supporting Information).

### 2.5 Neutrophil Isolation and Culture

Mouse peripheral blood neutrophils (PBNs) were isolated from the whole blood of 8‐week‐old male C57BL/6 mice using the Mouse Peripheral Blood Neutrophil Isolation Kit (Solarbio, Beijing, China). Blood was collected via cardiac puncture under anesthesia into anticoagulant tubes. The collected blood was diluted twice within 1 h and slowly added to the prepared separation system. The sample was then centrifuged at 1500 × g for 30 min at 25°C. Neutrophils, located at the interface of different density media, were carefully transferred to a new centrifuge tube. To remove excess red blood cells, an appropriate amount of lysis buffer was added. Neutrophils were washed three times with PBS and suspended in Mouse Peripheral Blood Neutrophil Complete Medium (CP‐M150, Pricella, Wuhan, China).

### 2.6 RNA Sequencing and Analysis

To investigate the impact of sepsis on the neutrophil transcriptome, we established a sepsis animal model through cecal ligation and puncture (CLP). Twelve hours after modeling, neutrophils were isolated from the peripheral blood of mice in the sham operation group (Sham group) and the CLP group, and total RNA was extracted. RNA sequencing was performed on the Illumina HiSeq X Ten platform (APExBIO Technology LLC, Shanghai, China). The raw RNA‐seq reads were saved in FASTQ format and quality control was evaluated using the FASTQC software. Low‐quality reads were removed using the FASTX‐Toolkit. High‐quality reads were mapped to the mouse reference genome (GRCm38/mm10) using TopHat (with parameters set as ‐G mouse_mRNA.gtf), and gene annotation and counting were performed using HTSeq. Differential expression analysis was completed using the DESeq2 package in R, and the fold change was calculated based on the ratio between the Sham group and the CLP group. When the p‐value was less than 0.05, the gene was considered significantly upregulated or downregulated. The heat map of differentially expressed genes was generated by the pheatmap package in R based on read counts. In addition, the clusterProfiler package in R was used to perform gene ontology (GO) enrichment analysis on differentially expressed genes, and the p‐values after multiple comparisons were corrected using the Benjamini‐Hochberg method. Upregulated and downregulated genes were classified according to the signature gene set.

### 2.7 Untargeted Metabolomics Analysis

The non‐targeted metabolomics detection experiment was conducted by APExBIO Technology, LLC (Shanghai, China). The specific steps are as follows:

1. Sample pretreatment: Take 100 µL of each sample and add 300 µL of methanol solution containing 5 µg mL^−1^ 2‐chloro‐L‐phenylalanine as an internal standard. Vortex for 1 min. Then centrifuge at 13,000 rpm for 10 min at 4°C. Transfer the supernatant to the injection vial for subsequent detection. Internal quality control (QC) samples are prepared by mixing equal amounts of each sample and passing through a 0.45 µm filter.
2. Liquid chromatography/mass spectrometry detection: Liquid chromatography‐mass spectrometry analysis was performed using an Agilent 1290 Infinity II ultra‐high performance liquid chromatography system coupled with an Agilent 6545 UHD Accurate‐Mass Q‐TOF/MS. The chromatographic column used was a Waters XSelect HSS T3 (2.5 µm, 100 × 2.1 mm). The mobile phase composition was as follows: Phase A was water with 0.1% formic acid; Phase B was acetonitrile with 0.1% formic acid. The flow rate was set at 0.4 mL min^−1^, the column temperature was 40° C, and the injection volume was 4 µL. The gradient elution conditions were: 0‐3 min, 20% B; 3‐9 min, 20%‐95% B; 9‐13 min, 95% B; 13‐13.1 min, 95%‐5% B; 13.1‐16 min, 5% B. The mass spectrometer operated in both positive and negative ion modes with the following optimized parameters: capillary voltages of 4.5 kV (positive mode) and 3.5 kV (negative mode), drying gas flow rates of 8 L min^−1^ (positive mode) and 10 L min^−1^ (negative mode), gas temperature of 325°C, nebulizer pressure of 20 psi, collision voltage of 120 V, extraction voltage of 45 V, and mass range of m/z 50‐1500.
3. Data analysis: The raw mass spectrometry data (wiff.scan files) were converted to MzXML files using ProteoWizard MSConvert and imported into the free XCMS software for peak selection. The parameters were set as follows: centWave m/z = 10 ppm, peak width = c(10, 60), pre‐filtering = c(10, 100). For peak grouping, bw = 5, mzwid = 0.025, minfrac = 0.5. CAMERA (a collection of metabolite profiling annotation algorithms) was used for isotope and adduct annotation. Only variables with more than 50% non‐zero measurements in at least one group were retained. Compound identification of metabolites was achieved by comparing the accurate m/z values (<10 ppm) and MS/MS spectra with an internal database (established based on existing standards).
4. Statistical analysis: After sum normalization, the data were subjected to principal component analysis (PCA) and orthogonal partial least squares discriminant analysis (OPLS‐DA) using the R package ropls. The robustness of the model was evaluated by sevenfold cross‐validation and response permutation tests. In the OPLS‐DA model, the variable importance in projection (VIP) value of each variable was calculated to indicate its contribution to classification. Student's t‐test was used to determine the significance of the difference between two independent samples. The criteria for screening significantly changed metabolites were VIP > 1 and p < 0.05. In addition, Pearson correlation analysis was conducted to determine the correlation between two variables.

### 2.8 Hematoxylin‐eosin (H&E) Staining

After euthanasia, the lungs, liver, and kidneys were collected from the mice and fixed overnight in 4% paraformaldehyde. The tissues were then dehydrated, embedded in paraffin, sectioned into 3 µm slices, and stained with hematoxylin and eosin (H&E). The sections were examined and photographed using optical microscopy.

### 2.9 Western Blotting Analysis

Protein samples were loaded onto 8%‐12% sodium dodecyl sulfate‐polyacrylamide gel electrophoresis (SDS‐PAGE) gels and electrophoresed at 90 V for 30 min, followed by a further 60 min at 120 V. Subsequently, the proteins were transferred onto polyvinylidene fluoride (PVDF) membranes. The PVDF membranes were blocked with 5% skim milk in TBST for 1 h and then incubated overnight at 4°C with primary antibodies. After incubation, the membranes were thoroughly washed and incubated with secondary antibodies. Detection was performed by chemiluminescence, and the band intensities were quantitatively analyzed using ImageJ software. The specific information of the primary antibodies used (including the concentrations) is provided in Table S2 (Supporting Information). The secondary antibodies were HRP‐conjugated Goat anti‐Rabbit IgG (H+L) (AS014) and HRP‐conjugated Goat anti‐Mouse IgG (H+L) (AS003), both purchased from ABclonal Technology (Wuhan, China), and were diluted at a ratio of 1:5000.

### 2.10 Co‐Immunoprecipitation

Protein interactions were assessed by immunoprecipitation. For HEK293T cells (used for exogenous immunoprecipitation), cells were transfected with the specified protein expression plasmid or control vector, while the specified PBNs were used for endogenous immunoprecipitation. After collecting these cells, they were lysed for 1 h in IP lysis buffer (Beyotime, Shanghai, China) containing a protease inhibitor mixture (MedChemExpress, MCE, Monmouth Junction, NJ, USA). The cell lysates were then centrifuged at 14,000 × g for 20 min at 4 °C. The supernatant was incubated with control IgG or the corresponding antibody and protein A/G magnetic beads (MCE, Monmouth Junction, NJ, USA) at 4 °C for 6 h for exogenous conditions and 12 h for endogenous conditions. The beads were washed three times with lysis buffer, and the immunoprecipitated complexes were resolved in SDS sample buffer for subsequent western blot analysis. The specific information of the primary antibodies (Flag, Myc, UBR5, PAD4) used is shown in Table S2 (Supporting Information), and they were diluted to a final concentration of 5–50 µg mL^−1^. The secondary antibody used was anti‐mouse IgG for IP (HRP) from Abcam (ab131368), diluted at a ratio of 1:5000.

### 2.11 Quantification of NETs

Detection and quantification of NETs in mouse serum and cell supernatants were performed using CitH3‐DNA or MPO‐DNA complex ELISA. Specifically, 96‐well plates were coated overnight at 4 °C with anti‐CitH3 antibody (ab5103, Abcam, USA) or anti‐MPO antibody (ab272101, Abcam, USA), followed by blocking with 2% BSA at room temperature for 2 h. Prior to the addition of the incubation buffer containing horseradish peroxidase‐conjugated anti‐DNA monoclonal antibody (Cell Death ELISAPLUS, 11774425001, Roche, Switzerland), the wells were washed three times. After a 2‐h incubation, the wells were washed three times again. ABTS peroxidase substrate was then added, and absorbance was measured at 405 nm using a microplate reader (Multiskanfc, Thermo Scientific).

### 2.12 ELISA

The levels of IL‐6, IL‐1β, TNF‐α were determined by ELISA kits (ABclonal, Shanghai,China) for human or mouse. Each sample was measured in duplicate.

### 2.13 RNA Isolation and Real‐Time Quantitative PCR (RT‐qPCR) Analysis

RNA was extracted from intestinal tissue using TRIzol reagent (15596026, Invitrogen) according to the manufacturer's instructions. The purity and concentration of the extracted RNA were measured using a NanoDrop spectrophotometer (Agilent Technologies, USA). Single‐stranded cDNA was synthesized using HiScript II QRT SuperMix for qPCR (R223‐01, Vazyme, China) following the provided protocol. PCR amplification and real‐time fluorescence quantification were performed using ChamQ SYBR Color qPCR Master Mix (Q431‐02, Vazyme, China). The relative expression of target genes was calculated using the 2^−ΔΔCT^ method, with ACTB as the reference gene. The qRT‐PCR primers used in this study were synthesized by Sangon Biotech (Shanghai) and are listed in Table S3 (Supporting Information).

### 2.14 Plasmids and Transfection

The plasmids used in this study were purchased from WZ Bioscience Inc. The plasmid construction steps in this study were as follows: First, the Acod1 fragment and the mutant fragments H159Q, L272Q, H277Y and N152S were amplified by PCR respectively, and specific primers were designed to add appropriate restriction enzyme sites at both ends of the fragments. Then, the amplified products and the pCDH‐CMV‐MCS‐EF1a‐Puro‐3flag vector with a C‐terminal Flag tag were digested with restriction enzymes to generate compatible sticky ends. Subsequently, the digested fragments were ligated to the vector using T4 DNA ligase to form recombinant plasmids. Similarly, the PAD4 fragment and the truncated bodies PAD4‐1‐118, PAD4‐119‐523 and PAD4‐524‐663 were amplified by PCR and restriction enzyme sites were added. After digestion, they were ligated into the pCDH‐CMV‐MCS‐COpGFP‐T2A‐Puro‐3flag vector with a C‐terminal FLAG tag. After construction, the plasmids were transformed into competent Escherichia coli cells, positive clones were screened by antibiotics, and colony PCR and sequencing were performed for verification.

The transfection steps are as follows: Transient transfection is performed using Lipofectamine 3000 transfection reagent. One day before transfection, cells are seeded into culture dishes at an appropriate density to reach 70‐90% confluence at the time of transfection. Plasmid DNA is diluted in serum‐free medium, and Lipofectamine 3000 is also diluted in serum‐free medium. Then, the two are mixed and incubated for 20 min. Next, the transfection complex is added dropwise to the culture dish containing cells, and the dish is gently shaken to ensure even distribution of the complex. After transfection, cells are further cultured in complete medium containing serum for 24–48 h to observe protein expression or conduct subsequent experiments.

### 2.15 Scanning Electron Microscopy (SEM)

Neutrophils from the designated group were spread on poly‐L‐lysine‐coated coverslips, fixed with 2% glutaraldehyde, and then post‐fixed by repeated incubation with 1% osmium tetroxide/1% tannic acid. The samples were dehydrated with increasing concentrations of ethanol and coated with 5‐nanometer carbon. Imaging was performed using a scanning electron microscope (USA‐FEI‐NOVA NANOSEM 230).

### 2.16 Statistical Analysis

Statistical analysis was performed using GraphPad Prism 8 software. For comparisons among multiple groups, one‐way ANOVA was used in combination with Dunnett's multiple comparison test; for comparisons between two groups affected by a single variable, Student's t‐test was employed; for comparisons among multiple groups involving two factors, two‐way ANOVA was utilized. Specific information on sample size (representing biological replicates) is provided in the corresponding figure legends. Survival analysis was conducted using the Kaplan‐Meier method, and survival curves were generated in Prism software through the Gehan‐Breslow‐Wilcoxon test. Data are presented as mean ± standard deviation or individual data points. All experiments were independently repeated at least three times, and the statistical significance threshold was set at *p* < 0.05. A *p*‐value > 0.05 indicates no significant difference, and the specific significance levels are marked with asterisks (*p < 0.05, **p < 0.01, ***p < 0.001), which were determined by the appropriate tests in GraphPad Prism.

## 3 Results

### 3.1 Acod1 is Closely Associated with Sepsis‐Related NETs

**Figure 1:**
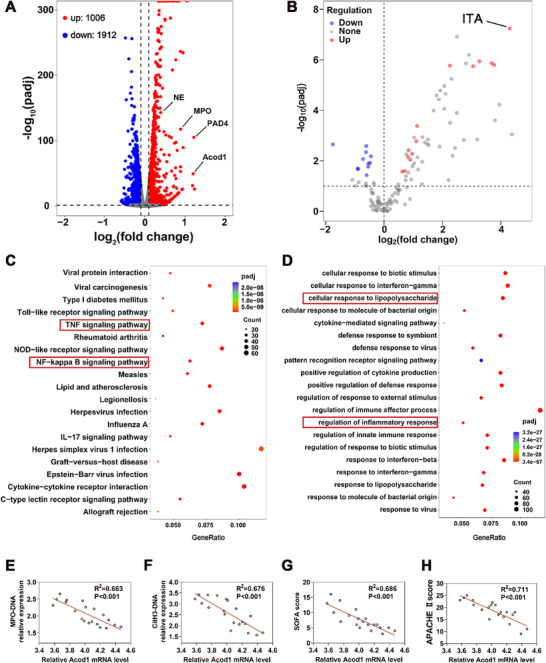
Acod1 is closely associated with sepsis‐related NETs. A) Volcano plot of differentially expressed genes in PBNs from C57BL/6 wild‐type mice between the CLP and Sham groups; B) Volcano plot of differentially expressed metabolites; C) GO enrichment analysis of differentially expressed genes; D) KEGG pathway enrichment analysis of differentially expressed genes; E,F) Pearson correlation analysis was conducted on the levels of MPO‐DNA and CitH3‐DNA in peripheral blood and the Acod1 mRNA level of patients with sepsis; G,H) Pearson correlation analysis was carried out on the SOFA score and APACHE II score of patients with sepsis and the Acod1 mRNA level. n=3 per group (A‐D), n=20 per group (E‐H). Student's *t*‐test is used to compare two groups of data affected by a single variable. For the comparison of multiple groups of data, one‐way ANOVA is adopted, and Dunnett's multiple comparisons test is used for post hoc analysis. All data are presented as mean ± standard deviation. Differences were considered statistically significant at *p < 0.05, **p < 0.01, and ***p < 0.001.

A sepsis animal model was established using CLP. Twelve hours post‐modeling, samples were collected from the PBNs of mice in both the Sham and CLP groups for transcriptomic and metabolomic sequencing. Transcriptomic sequencing results showed that, compared to the Sham group, the levels of NETs‐related genes such as PAD4, MPO, and NE were significantly upregulated in the neutrophils of the CLP group. Additionally, the expression level of Acod1 was also markedly increased in the CLP group (**Figure** **1**A). Metabolomic sequencing data indicated that the level of ITA was significantly elevated in the neutrophils of the CLP group compared to the Sham group (Figure 1B). Further GO enrichment analysis revealed significant enrichment of immune and inflammatory biological processes, such as inflammatory response regulation, in the neutrophils of the CLP group compared to the Sham group (Figure 1C). KEGG pathway enrichment analysis showed significant enrichment of immune and inflammatory signaling pathways, including TNF signaling and NF‐kappa B signaling pathways, in the neutrophils of the CLP group compared to the Sham group (Figure 1D). Analysis of the RNAseq dataset (GSE154918) revealed that the Acod1 gene was significantly upregulated in clinical sepsis patient samples compared to healthy controls (Figure S1A, Supporting Information). Further analysis showed a correlation between Acod1 gene levels and PAD4 and NE gene levels in sepsis patients (Figure S1B,C, Supporting Information).

To further verify the conclusions drawn from the transcriptome and metabolome sequencing and bioinformatics analysis, this study included confirmed patients meeting the sepsis 3.0 criteria and healthy volunteers at the clinical level. We detected the expression level of Acod1 mRNA in peripheral blood neutrophils of healthy volunteers and sepsis patients, and determined the concentration of pro‐inflammatory factor TNF‐α in peripheral blood, as well as the content of NETs' characteristic components CitH3‐DNA and MPO‐DNA (Figure S2A–E, Supporting Information). The experimental results showed that compared with healthy individuals, the levels of TNF‐α and NETs in the peripheral blood of sepsis patients were significantly increased. Furthermore, we conducted a correlation analysis of the Acod1 mRNA level in the peripheral blood of sepsis patients and the levels of NETs markers. The results indicated that there was a significant negative correlation between the two (Figure 1E,F), suggesting that Acod1 might play an important role in regulating the generation of NETs. Notably, the Acod1 mRNA level also showed a negative correlation trend with the TNF‐α level (Figure S2F, Supporting Information). To further explore the clinical significance of Acod1, we analyzed the relationship between its mRNA level and the severity of sepsis. The Sequential Organ Failure Assessment (SOFA) score and Acute Physiology and Chronic Health Evaluation II (APACHE II) score systems, which are commonly used in clinical practice, were adopted. The results showed that the expression level of Acod1 mRNA was significantly negatively correlated with the severity of sepsis (Figure 1G,H). These results suggest that Acod1/ITA plays a crucial role in the immune regulation and inflammatory response of neutrophils in sepsis and is closely associated with the formation of NETs.

### 3.2 Acod1 Knockout Exacerbated the Progression of Sepsis and Increased Mortality

**Figure 2:**
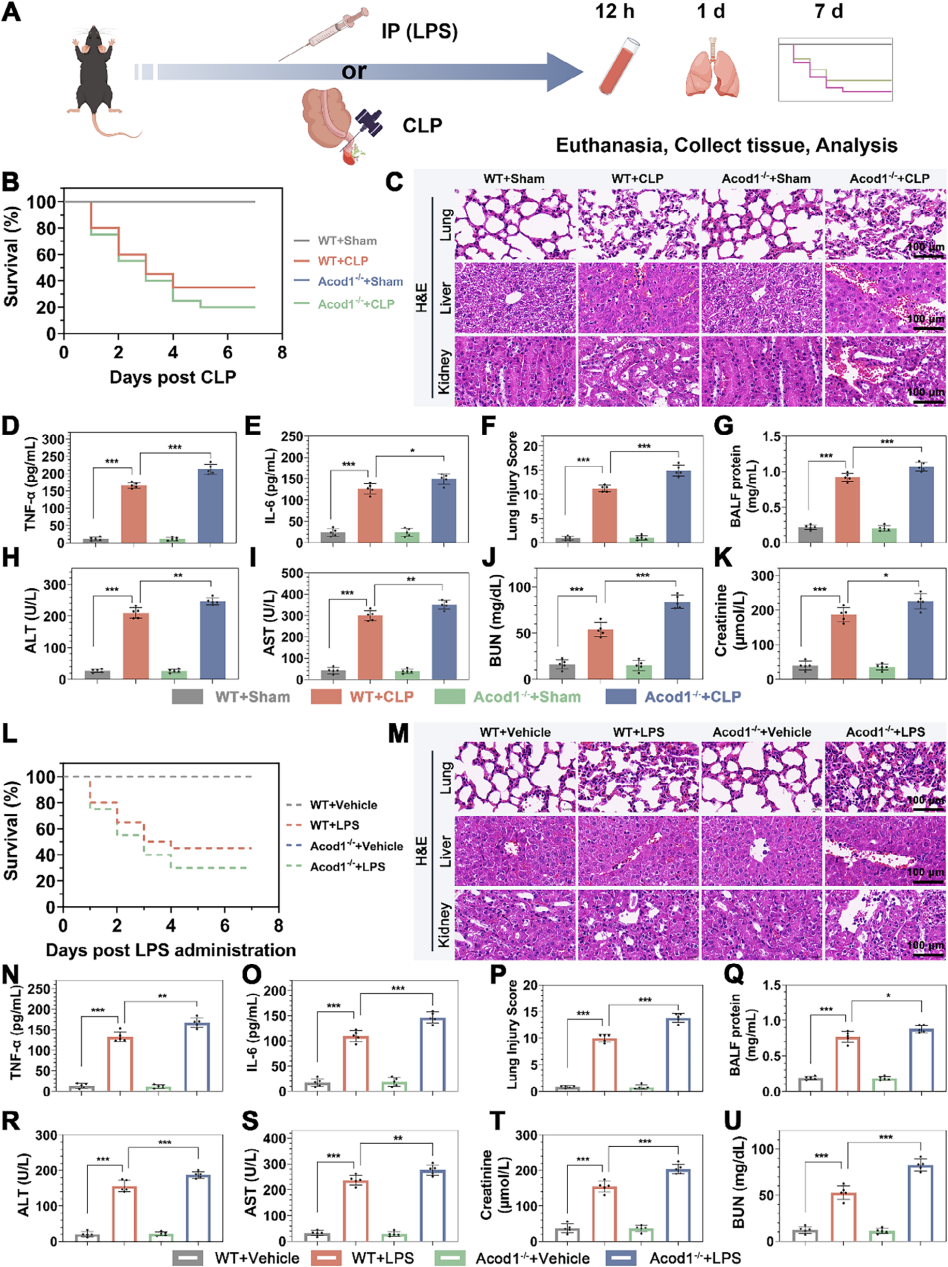
Acod1 knockout exacerbated the progression of sepsis and increased mortality. A) Diagram of mouse modeling and treatment. After CLP modeling, B) 7‐day survival rates of mice; C) HE staining results of lung, liver, and kidney tissues (scale bar = 100 µm); D,E) Levels of pro‐inflammatory cytokines TNF‐α and IL‐6 in peripheral blood detected by ELISA; F,G) Assessment of lung damage using lung tissue pathology scores and protein content in BALF; H,I) Liver damage assessed by alanine aminotransferase (ALT) and aspartate aminotransferase (AST) levels; J,K) Kidney damage assessed by serum creatinine (Cr) and blood urea nitrogen (BUN) levels. After LPS‐induced sepsis modeling, L) 7‐day survival rates of mice; M) HE staining results of lung, liver, and kidney tissues (scale bar = 100 µm); N,O) Levels of pro‐inflammatory cytokines TNF‐α and IL‐6 in peripheral blood detected by ELISA; P,Q) Assessment of lung damage using lung tissue pathology scores and protein content in BALF; R,S) Liver damage assessed by ALT and AST levels; T,U) Kidney damage assessed by serum Cr and BUN levels. n=20 per group (B and L), n=5 per group (C‐K and M‐U). Student's *t*‐test is used to compare two groups of data affected by a single variable. For the comparison of multiple groups of data, one‐way ANOVA is adopted, and Dunnett's multiple comparisons test is used for post hoc analysis. All data are presented as mean ± standard deviation. Differences were considered statistically significant at *p < 0.05, **p < 0.01, and ***p < 0.001.

WB analysis confirmed successful knockout of Acod1 in PBNs from Acod1^‐/‐^ mice (Figure S3A, Supporting Information), with a significant reduction in ITA levels in PBNs following Acod1 knockout (Figure S3B, Supporting Information). To thoroughly investigate the role of Acod1 in sepsis, we established two animal models: the CLP sepsis model and the LPS‐induced sepsis model (**Figure** **2**A). Seven days after establishing the CLP sepsis model, the survival rate of Acod1^‐/‐^ mice was significantly lower than that of WT mice (Figure 2B). HE staining results showed that significant pathological changes occurred in the lung, liver, kidney and heart tissues of CLP‐induced mice, including edema, hemorrhage, abnormal tissue structure and inflammatory cell infiltration. Notably, compared with wild‐type (WT) mice, the lung, liver kidney and heart tissues of Acod1 ‐ / ‐ mice suffered more severe damage (Figure 2C; Figure S4A, Supporting Information). ELISA results showed that levels of TNF‐α, IL‐1β, and IL‐6 in the peripheral blood of Acod1^‐/‐^ mice were significantly higher than in WT mice (Figure 2D,E; Figure S4C, Supporting Information). HE staining lung damage pathology scores revealed significantly greater lung tissue damage in Acod1^‐/‐^ mice compared to WT mice (Figure 2F), and protein levels in BALF were also higher in Acod1^‐/‐^ mice (Figure 2G). Blood biochemical tests showed increased liver and kidney dysfunction in Acod1^‐/‐^ mice compared to WT mice, as evidenced by higher ALT, AST, creatinine, and urea nitrogen levels (Figure 2H–K). These findings were validated in the LPS‐induced sepsis model, where Acod1^‐/‐^ mice exhibited reduced 7‐day survival rates and more severe peripheral blood inflammation and organ damage compared to WT mice, consistent with the observations in the CLP model (Figure 2L–U; Figure S4B,D, Supporting Information). These results indicate that Acod1 plays a crucial role in the pathogenesis of sepsis.

### 3.3 Acod1 Knockout Promotes NETs Formation both In Vivo and In Vitro

**Figure 3:**
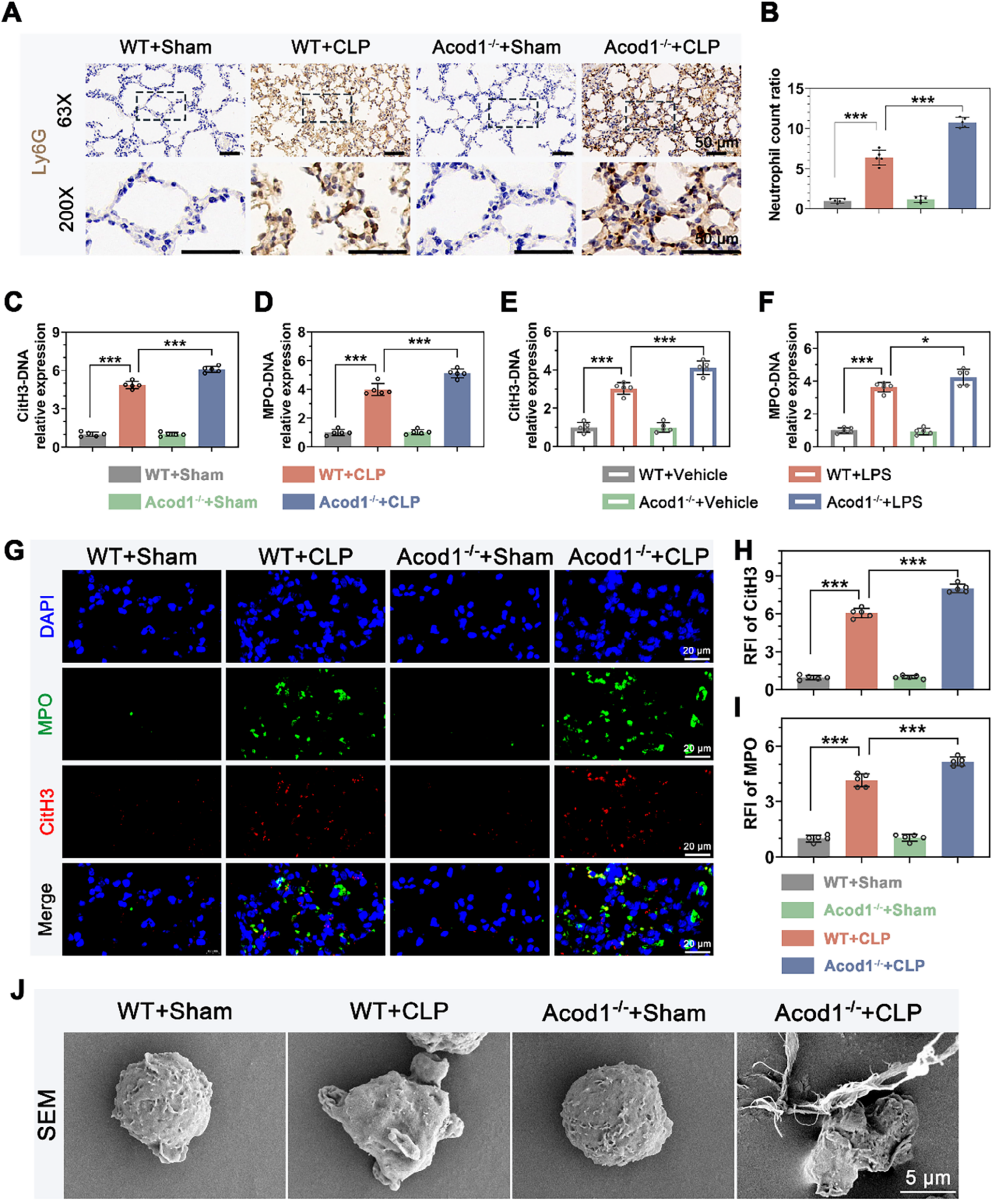
Acod1 knockout promotes NETs formation. Immunohistochemical staining of the neutrophil marker Ly6G in mouse lung tissue (A) shows representative images and (B) quantitative analysis (scale bar = 50 µm). C–F) display the concentration levels of NETs markers CitH3‐DNA and MPO‐DNA complexes in the peripheral blood of mice. G) shows representative images and (H,I) quantitative analysis of NETs markers (CitH3 and MPO) in mouse lung tissue (scale bar = 20 µm). J) presents representative scanning electron microscopy images of PBN cell morphology in mouse peripheral blood (scale bar = 5 µm). n = 5 per group. Student's *t*‐test is used to compare two groups of data affected by a single variable. For the comparison of multiple groups of data, one‐way ANOVA is adopted, and Dunnett's multiple comparisons test is used for post hoc analysis. All data are presented as mean ± standard deviation. Differences were considered statistically significant at *p < 0.05, **p < 0.01, and ***p < 0.001.

We investigated the impact of Acod1 on NETs formation both in vivo and in vitro. Immunohistochemical staining of lung tissue revealed that after CLP modeling, the number of Ly6G‐positive cells in the lung tissue of Acod1^‐/‐^ mice was significantly higher than in WT mice, indicating that Acod1 knockout increased the number of activated neutrophils in CLP mice (**Figure** **3**A,B). ELISA analysis showed that after CLP modeling, the levels of CitH3‐DNA and MPO‐DNA complexes in the peripheral blood of Acod1^‐/‐^ mice were significantly higher than those in WT mice (Figure 3C,D). Similar results were obtained in the peripheral blood of mice in the LPS‐induced sepsis model, with elevated levels of CitH3‐DNA and MPO‐DNA complexes (Figure 3E,F). Immunofluorescence imaging of lung tissue showed that after CLP modeling, the fluorescence intensity of CitH3 and MPO in Acod1^‐/‐^ mice was significantly higher than in WT mice (Figure 3G–I). Scanning electron microscopy of PBNs isolated from the peripheral blood of mice revealed a substantial amount of extracellular net‐like structures surrounding PBNs in Acod1^‐/‐^ mice, far exceeding that in WT mice (Figure 3J). These results indicate that Acod1 knockout leads to a significant increase in NETs production in septic mice.

Subsequently, in vitro experiments using mouse PBNs and dHL60 cells (a human promyelocytic leukemia cell line with neutrophil‐like differentiation) were conducted to assess the effect of Acod1 on inflammatory factor release and NETs formation. The results showed that Acod1 knockout significantly increased the release of TNF‐α, IL‐1β, and IL‐6 from PBNs and dHL60 cells (Figure S5A–C,F–H), and the levels of CitH3‐DNA and MPO‐DNA complexes in the culture supernatants of PBNs and dHL60 cells were also significantly elevated (Figure S5D,E,I,J). These findings indicate that Acod1 knockout leads to a significant increase in NETs formation in cultured neutrophils.

### 3.4 Acod1 Regulates NETosis Through the Itaconate Pathway

**Figure 4:**
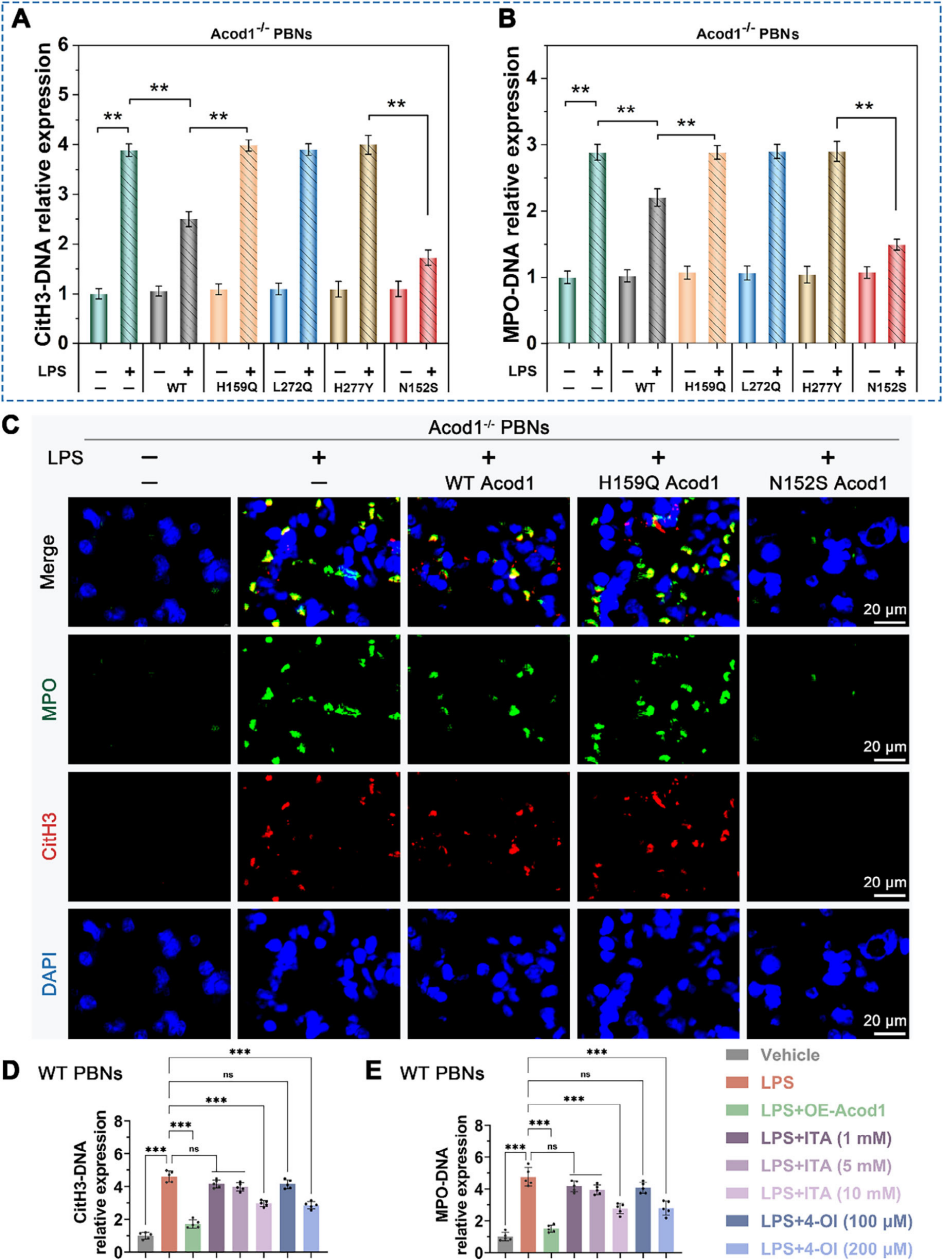
Acod1 regulates NETosis through the itaconate pathway. In Acod1^‐/‐^ PBNs cells, a series of Acod1 plasmids and mutant plasmids were transfected. A,B) The concentrations of NETs markers CitH3‐DNA and MPO‐DNA complexes in the cell supernatants were detected by ELISA. C) The fluorescence intensity of CitH3 and MPO was assessed by immunofluorescence (scale bar = 20 µm). In WT PBNs cells, overexpression of Acod1 or addition of exogenous ITA was performed. D,E) The concentrations of NETs markers CitH3‐DNA and MPO‐DNA complexes in the cell supernatants were measured by ELISA. n = 5 per group. Student's *t*‐test is used to compare two groups of data affected by a single variable. For the comparison of multiple groups of data, one‐way ANOVA is adopted, and Dunnett's multiple comparisons test is used for post hoc analysis. All data are presented as mean ± standard deviation. Differences were considered statistically significant at *p < 0.05, **p < 0.01, and ***p < 0.001.

Previous studies showed that amino acids 159, 272, and 277 of the Acod1 protein were identified as active functional sites directly involved in the production of itaconate.^[^ ^22^ ^]^ Additionally, the missense polymorphism Asn152Ser in Acod1 significantly increased itaconate production compared to wild‐type Acod1.^[^ ^23^ ^]^ To determine whether Acod1 regulates NETosis (neutrophil extracellular trap formation) directly through the protein or via the itaconate pathway, this study transfected wild‐type Acod1 (WT Acod1) and a series of Acod1 mutant plasmids into Acod1 knockout PBNs cells. ELISA results showed that compared with the control group transfected with the empty vector, the levels of CitH3‐DNA and MPO‐DNA in Acod1^‐/‐^ PBNs cells transfected with the mutant plasmids (H159Q, L272Q and H277Y) that do not produce itaconic acid did not change significantly. In contrast, the levels of CitH3‐DNA and MPO‐DNA in cells transfected with the wild‐type Acod1 plasmid or the N152S mutant plasmid that overexpresses itaconic acid were significantly reduced (**Figure** **4**A,B). Notably, the decrease in CitH3‐DNA and MPO‐DNA levels caused by the N152S mutant was more pronounced than that by WT. The results of immunofluorescence detection of CitH3 and MPO were consistent with these findings, further supporting these observations (Figure 4C). Thus, it can be seen that in Acod1^‐/‐^ PBNs, transfection of the three Acod1 mutant plasmids (H159Q, L272Q and H277Y) that do not produce itaconic acid does not affect NETosis; while transfection of the N152S mutant plasmid that overexpresses itaconic acid can significantly inhibit NETosis. Therefore, we infer that Acod1 regulates NETosis indirectly through the itaconic acid pathway rather than directly through its own protein.

Subsequently, we compared the effects of Acod1 overexpression in PBNs, exogenous itaconate (ITA) addition and itaconate derivative 4‐octyl itaconate (4‐OI) on NETosis.^[^ ^24^ ^]^ The experimental results showed that when the itaconate concentration reached 10 mM, it could significantly inhibit the generation of LPS‐induced CitH3‐DNA and MPO‐DNA complexes (Figure 4D,E). Additionally, 4‐OI with better cell membrane permeability also exhibited a significant inhibitory effect at a concentration of 200 µM. However, compared with Acod1 overexpression, the inhibitory effects of exogenous itaconate or 4‐OI on the generation of CitH3‐DNA and MPO‐DNA complexes were relatively weak (Figure 4D,E). Notably, both 10 mM of itaconate and 200 µM of 4‐OI could effectively alleviate the excessive generation of CitH3‐DNA and MPO‐DNA complexes caused by Acod1 gene knockout (Figure S6A,B). In summary, our research results indicate that Acod1 regulates the formation of NETs through the itaconate metabolic pathway rather than its direct protein action.

**Figure 5:**
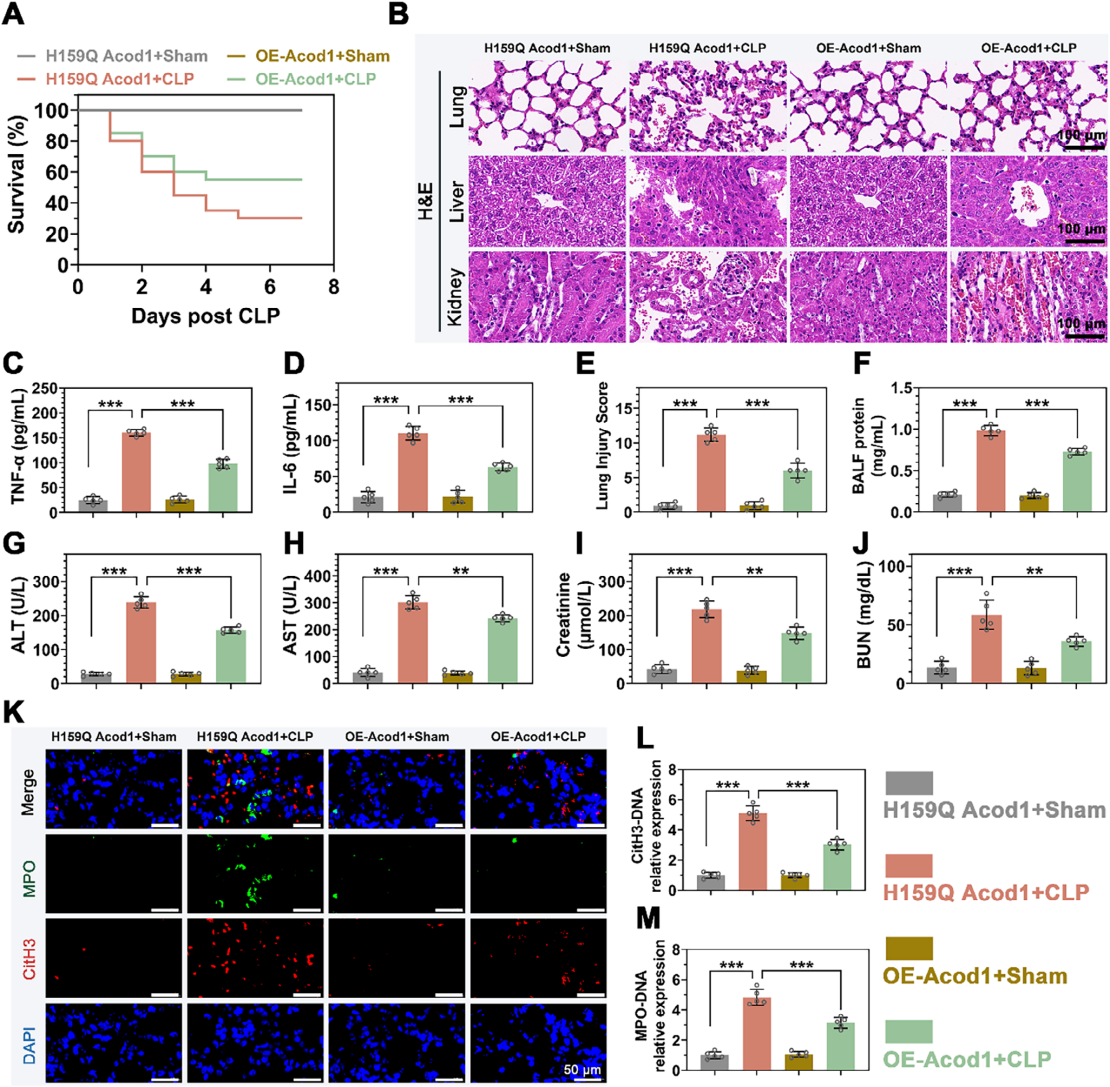
Acod1 regulates NETosis through the ITA pathway, thereby providing protective effects in sepsis. After CLP modeling, A) 7‐day survival rate of mice; B) HE staining of lung, liver, and kidney tissues of mice (scale bar = 100 µm); C,D) detection of pro‐inflammatory cytokines TNF‐α and IL‐6 levels in the peripheral blood of mice by ELISA; E,F) assessment of lung injury by pathological damage scoring of lung tissue and protein content in BALF; G,H) evaluation of liver injury by detecting serum levels of ALT and AST; I,J) evaluation of kidney injury by measuring serum creatinine and blood urea nitrogen levels; K) representative immunofluorescence images of MPO and CitH3 in mouse lung tissues (scale bar = 50 µm); L,M) detection of the concentrations of NETs markers CitH3‐DNA and MPO‐DNA complexes in the peripheral blood of mice. For (A), n = 20 per group; for (B‐M), n = 5 per group. Student's *t*‐test is used to compare two groups of data affected by a single variable. For the comparison of multiple groups of data, one‐way ANOVA is adopted, and Dunnett's multiple comparisons test is used for post hoc analysis. All data are presented as mean ± standard deviation. Differences were considered statistically significant at *p < 0.05, **p < 0.01, and ***p < 0.001.

Further investigation confirmed the impact of Acod1/ITA on sepsis and NETosis. Compared to the H159Q Acod1 group, which does not produce ITA, the OE‐Acod1 group with overexpressed ITA showed a significant increase in 7‐day survival rate (**Figure** **5**A), as well as a notable reduction in systemic inflammatory response and organ damage (Figure 5B–J; Figure S7A, Supporting Information). The levels of NETs were also reduced accordingly (Figure 5K–M; Figure S7B,C, Supporting Information). These results indicate that Acod1 regulates NETosis through the ITA pathway, thereby providing protective effects in sepsis.

### 3.5 Acod1 Promotes the Degradation of the NETosis Hub Protein PAD4

We investigated how Acod1/ITA regulates NETosis. PAD4, MPO and NE are the core proteins involved in the formation of NETs. NETosis is the process by which neutrophils form and release NETs through a specific programmed cell death pathway. During this process, neutrophils first sense endogenous and exogenous stimuli, produce ROS, and then activate PAD4.^[^ ^25^ ^]^ Subsequently, PAD4 catalyzes the citrullination of histone arginine residues, promoting chromatin decondensation and release into the cytoplasm. The decondensed chromatin combines with cytoplasmic granular enzymes (such as MPO and NE) and is eventually released extracellularly to form NETs. Specifically, MPO binds to chromatin and activates NE in azurophilic granules.^[^ ^26^ ^]^ NE breaks down cytoplasmic actin, translocates to the nucleus and cleaves histones, further promoting chromatin decondensation. The coordinated action of these core proteins is crucial for NET formation.^[^ ^10^ ^]^ Therefore, we are particularly interested in whether Acod1/ITA regulates the mRNA and protein expression levels of PAD4, MPO and NE.

**Figure 6:**
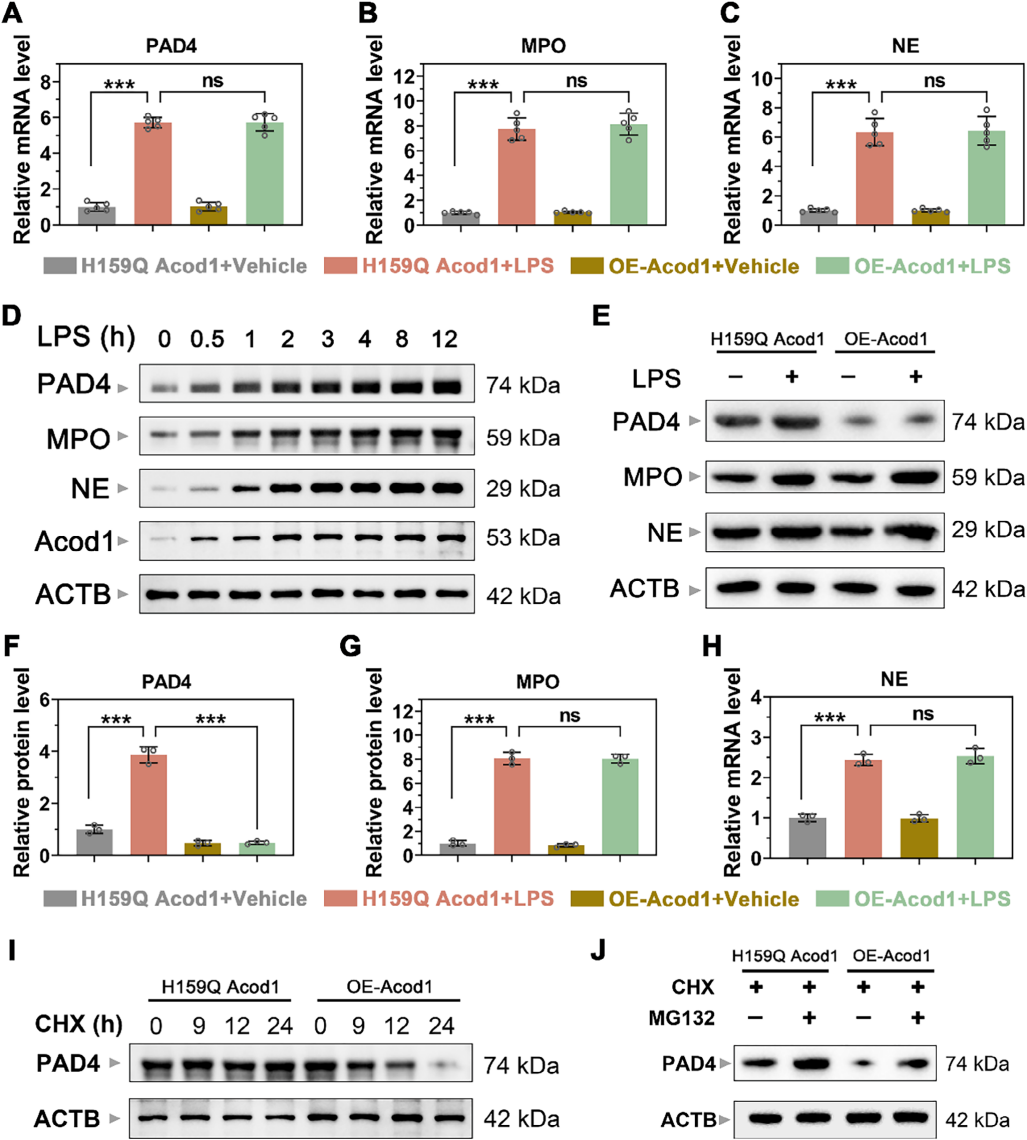
Acod1/ITA promotes the degradation of the NETosis hub protein PAD4. A–C) qRT‐PCR was used to measure the mRNA levels in cells transfected with H159Q Acod1 plasmid, which does not express ITA, and OE‐Acod1 plasmid, which overexpresses ITA, under LPS stimulation. D) Western blot was used to detect the protein levels of PAD4, MPO, NE, and Acod1 in PBNs at different time points of LPS stimulation. E–H) Western blot was employed to measure and quantify the protein levels of PAD4, MPO, and NE in cells transfected with H159Q Acod1 and OE‐Acod1 plasmids under LPS stimulation. I) Western Blot was used to assess the PAD4 protein levels in PBNs cells under 20 µg mL^−1^ CHX treatment. J) PBNs cells were treated with CHX and stimulated with MG132 (10 µM) for 9 h. The PAD4 protein was determined by Western blotting. n = 5 per group (A‐C), n = 3 per group (D–J). Student's *t*‐test is used to compare two groups of data affected by a single variable. For the comparison of multiple groups of data, one‐way ANOVA is adopted, and Dunnett's multiple comparisons test is used for post hoc analysis. All data are presented as mean ± standard deviation. Differences were considered statistically significant at *p < 0.05, **p < 0.01, and ***p < 0.001.

Western blotting (WB) analysis confirmed successful overexpression of Acod1 (Figure S8A, Supporting Information). PCR results indicated that under LPS stimulation, the transcription levels of the core NET formation proteins PAD4, MPO, and NE were significantly increased. However, compared to the H159Q Acod1 group that does not produce ITA, the transcription levels of PAD4, MPO, and NE did not show significant differences in the OE‐Acod1 group that overexpresses ITA (**Figure** **6**A–C). Further WB analysis revealed a significant increase in the protein levels of PAD4, MPO, and NE under LPS stimulation (Figure 6D). Nonetheless, compared to the H159Q Acod1 group, the protein levels of MPO and NE did not show significant differences in the OE‐Acod1 group, whereas the PAD4 protein level was decreased (Figure 6E–H).

Since Acod1/ITA did not significantly affect the transcriptional level of PAD4 but reduced its protein level, we speculated that Acod1/ITA might function by regulating the degradation of PAD4. The ubiquitin‐proteasome system (UPS) and autophagy are the main pathways for intracellular protein degradation.^[^ ^27^ ^]^ To explore the specific mechanism by which Acod1/ITA promotes the degradation of PAD4, we evaluated the dynamic changes of PAD4 in PBNs after treatment with the protein synthesis inhibitor cycloheximide (CHX) for 0, 9, 12, and 24 h. The results showed that compared with the H159Q Acod1 group, the OE‐Acod1 group significantly accelerated the degradation of PAD4 (Figure 6I). MG132 is a potent and specific proteasome inhibitor that reversibly blocks the enzymatic activity of the proteasome. Further studies found that the addition of MG132 could reverse the degradation of PAD4 in the OE‐Acod1 group (Figure 6J). In contrast, no significant changes in the degradation of PAD4 protein were observed in mouse PBNs cells after treatment with the autophagy inhibitor chloroquine (CQ) (Figure S9, Supporting Information). These results suggest that Acod1/ITA may mainly promote the rapid degradation of PAD4 through the ubiquitin‐proteasome pathway.

### 3.6 Acod1 Regulates NETosis by Promoting the K48‐Linked Ubiquitin‐Dependent Degradation of PAD4

**Figure 7:**
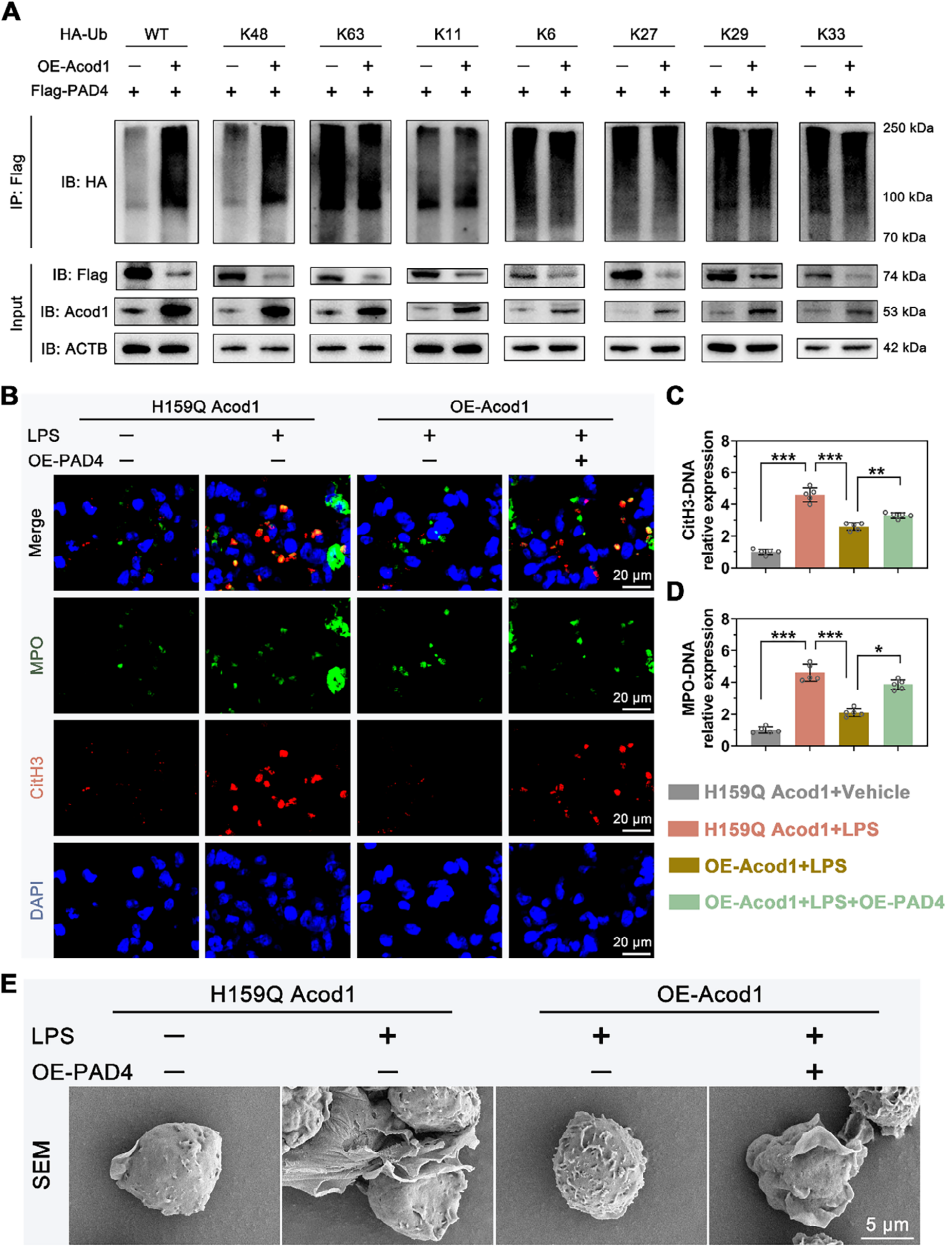
Acod1/ITA regulates NETosis by promoting the K48‐linked ubiquitin‐dependent degradation of PAD4. A) Ubiquitination detection of PAD4 protein. B) Representative immunofluorescence images of CitH3 and MPO in mouse PBNs (scale bar = 20 µm). C,D) Concentration levels of CitH3‐DNA and MPO‐DNA complexes in the culture supernatants of mouse PBNs cells. E) Scanning electron microscopy image of mouse PBNs cells (scale bar = 5 µm). Sample sizes: n=3 for (A), n=5 for (B‐E). Student's *t*‐test is used to compare two groups of data affected by a single variable. For the comparison of multiple groups of data, one‐way ANOVA is adopted, and Dunnett's multiple comparisons test is used for post hoc analysis. All data are presented as mean ± standard deviation. Differences were considered statistically significant at *p < 0.05, **p < 0.01, and ***p < 0.001.

Western blot was used to examine the effect of Acod1 on PAD4 ubiquitination levels. The results showed that Acod1 promotes overall ubiquitination of PAD4, predominantly through K48‐linked ubiquitination (**Figure** **7**A). To determine the critical role of PAD4 in Acod1/ITA regulation of NETosis, a rescue experiment using PAD4 overexpression plasmid was performed. Western blot results demonstrated successful overexpression of PAD4 in PBNs transfected with the PAD4 overexpression plasmid (Figure S8B, Supporting Information). Immunofluorescence, Western blot, and scanning electron microscopy results indicated that overexpression of PAD4 can block the reduction in fluorescence intensity of CitH3 and MPO in the OE‐Acod1 group (Figure 7B), reverse the reduction of CitH3‐DNA and MPO‐DNA complex levels in the culture supernatants of PBNs induced by OE‐Acod1 (Figure 7C,D), and reverse the reduction in extracellular net‐like structure release from PBNs in the OE‐Acod1 group (Figure 7E). These results suggest that Acod1 regulates NETs formation by affecting PAD4 protein levels. Thus, overexpression of Acod1 promotes K48‐linked ubiquitination of PAD4, leading to PAD4 degradation via the ubiquitin‐proteasome pathway, and thereby regulates NETosis.

### 3.7 Acod1 Promotes PAD4 Degradation in a UBR5‐Dependent Manner

**Figure 8:**
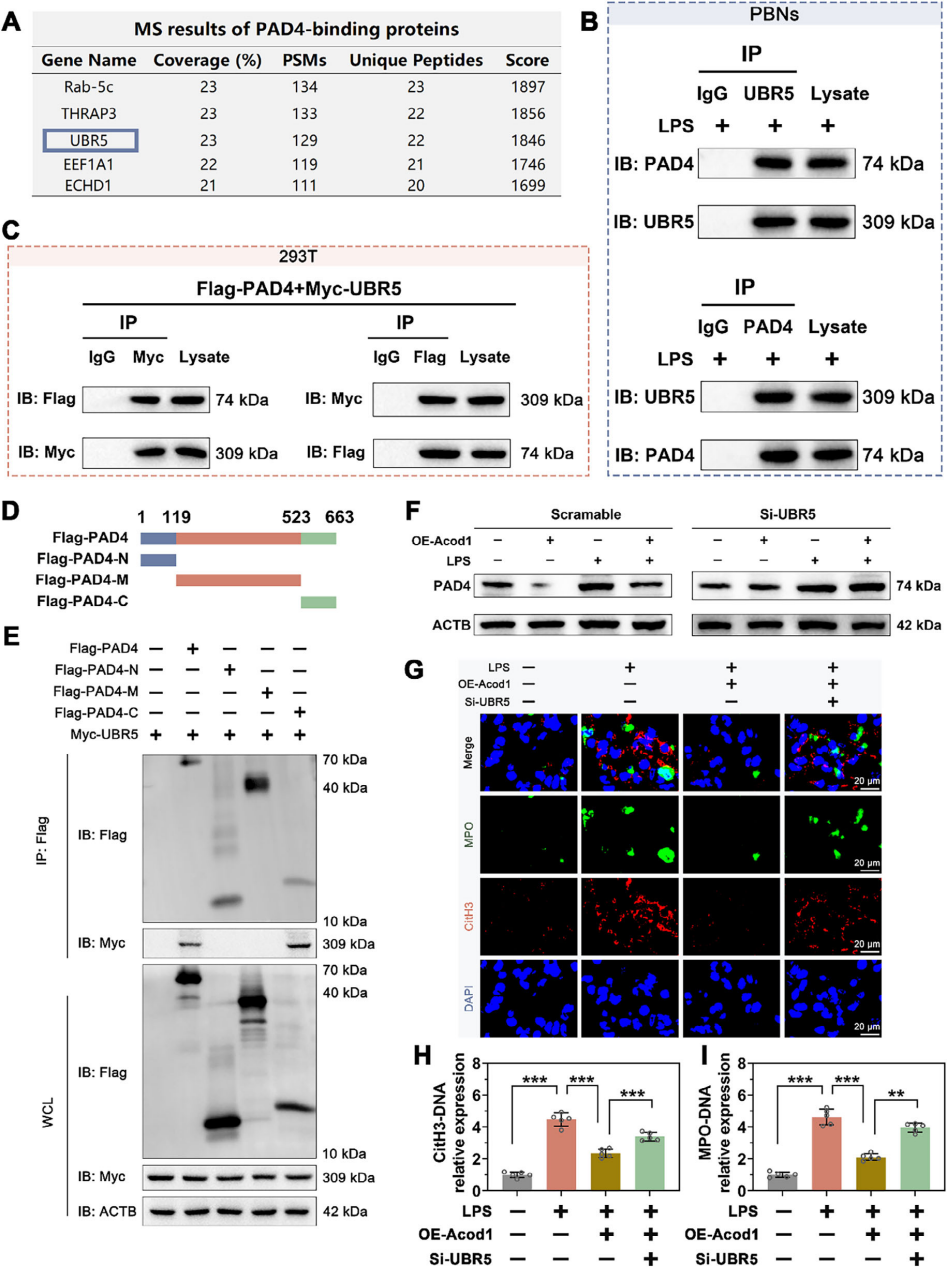
Acod1 promotes PAD4 degradation in a UBR5‐dependent manner. A) Proteomic analysis identifies proteins that bind to PAD4 in PBNs cells, showing the proteins with the highest binding scores. B,C) Co‐IP assays demonstrate the interaction between PAD4 and UBR5. D) Schematic representation of the full‐length PAD4 protein and its truncated domains. E) Immunoprecipitation assays assess the interaction between full‐length PAD4 (aa 1–663), PAD4‐N (aa 1–118), PAD4‐M (aa 119–522), or PAD4‐C (aa 523–663) with UBR5. F) Effects of UBR5 knockdown on PAD4 protein levels in PBNs cells are assessed. G) Immunofluorescence analysis measures the fluorescence intensity of CitH3 and MPO in the supernatant of PBNs cell cultures (scale bar = 20 µm). H,I) Concentration levels of CitH3‐DNA and MPO‐DNA complexes in the supernatant of PBNs cell cultures are determined. n=3 per group (B, C, E, and F); n=5 per group (H‐I). Student's *t*‐test is used to compare two groups of data affected by a single variable. For the comparison of multiple groups of data, one‐way ANOVA is adopted, and Dunnett's multiple comparisons test is used for post hoc analysis. All data are presented as mean ± standard deviation. Differences were considered statistically significant at *p < 0.05, **p < 0.01, and ***p < 0.001.

The ubiquitin‐proteasome pathway is a crucial intracellular mechanism for selective protein degradation, where E3 ubiquitin ligases play a key role in substrate specificity. To investigate the regulatory mechanism of Acod1 on PAD4 ubiquitination, we utilized proteomic analysis to identify proteins that interact with PAD4 in PBNs cells. The results showed that among the ubiquitination‐related proteins that bind to PAD4, the E3 ubiquitin ligase UBR5 had the highest binding score (**Figure** **8**A). To further explore the interaction between PAD4 and UBR5, we treated PBNs cells with 100 ng mL^−1^ LPS and performed co‐immunoprecipitation (Co‐IP) assays. The results demonstrated that PAD4 interacts with UBR5 within PBNs cells (Figure 8B). Subsequently, we transfected HEK293T cells with plasmids tagged with Flag‐PAD4 and Myc‐UBR5, and Co‐IP results confirmed the interaction between PAD4 and UBR5 (Figure 8C).

We further identified the specific protein domains responsible for the interaction between PAD4 and UBR5. We expressed full‐length PAD4 and a series of PAD4 truncation mutants in 293T cells. The experiment revealed that the interaction between the amino acid residues 523 to 663 (PAD4‐C) and UBR5 was stronger than with full‐length PAD4, whereas residues 1 to 118 (PAD4‐N) and residues 119 to 522 (PAD4‐M) did not interact with UBR5 (Figure 8D,E). These results indicate that UBR5 primarily interacts with the PAD4‐C (aa523‐663) domain of PAD4.

To determine the critical role of UBR5 in Acod1/ITA regulation of NETosis, we conducted a rescue experiment with UBR5 knockdown. Western blotting results showed successful knockdown of UBR5 in PBNs (Figure S8C, Supporting Information). Further experiments revealed that UBR5 knockdown could reverse the PAD4 protein degradation induced by OE‐Acod1 (Figure 8F), block the decrease in fluorescence intensity of CitH3 and MPO caused by OE‐Acod1 (Figure 8G), and reverse the reduction of CitH3‐DNA and MPO‐DNA complex levels in the culture supernatants of PBNs induced by OE‐Acod1 (Figure 8H,I). These results suggest that Acod1/ITA promotes PAD4 degradation in a UBR5‐dependent manner, thereby regulating NET formation.

### 3.8 Acod1 Enhances the Enzymatic Activity of UBR5 Through Alkylation Modification, thereby Promoting the Ubiquitination and Degradation of PAD4

**Figure 9:**
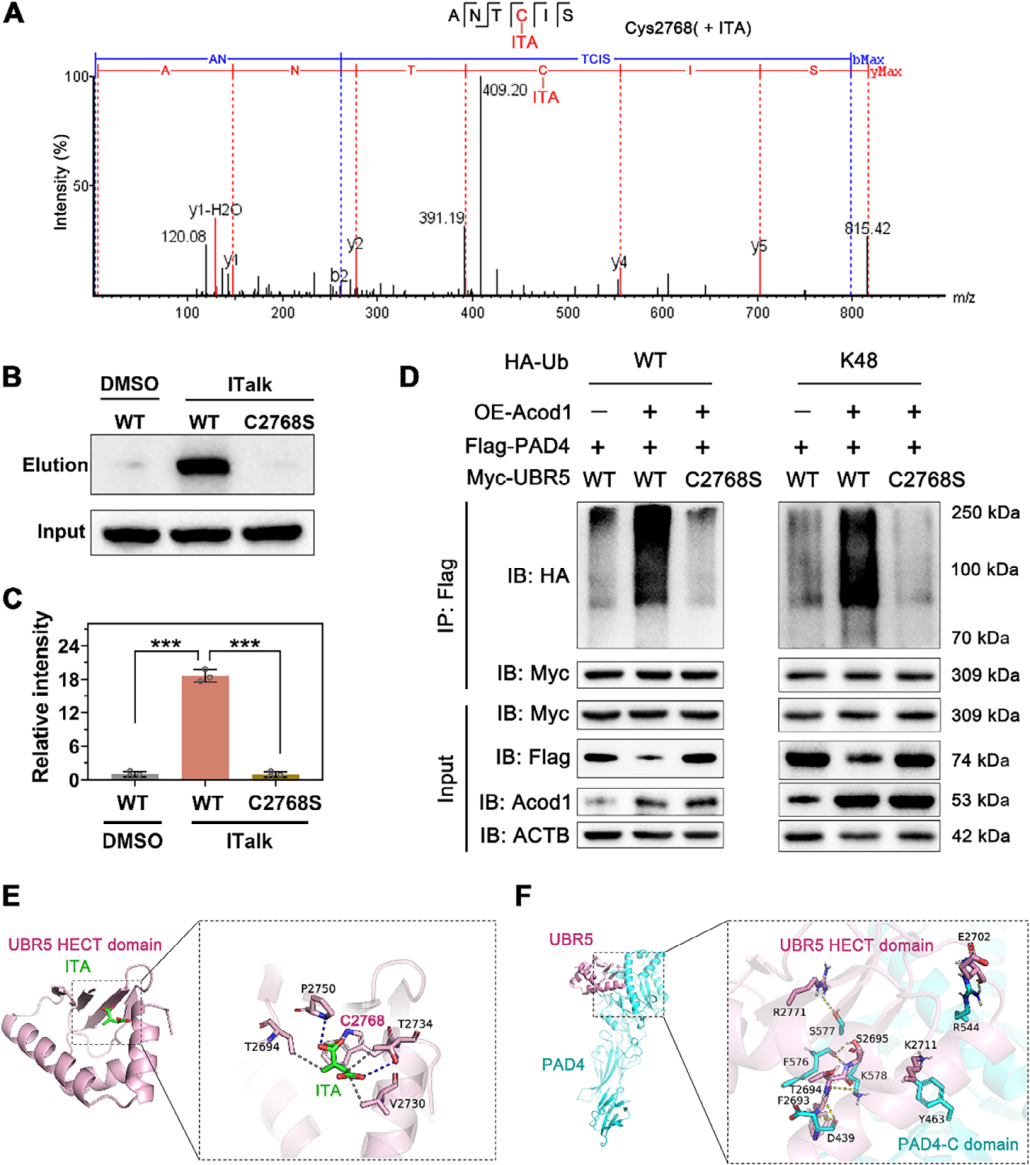
Acod1 enhances the enzymatic activity of UBR5 through alkylation modification, thereby promoting the ubiquitination and degradation of PAD4. A) Proteomic analysis revealed alkylation modification of UBR5 protein; B,C) PBNs cells were infected with wild‐type UBR5 (referred to as WT) and the indicated mutants. After 2 h of incubation with either ITalk probe or vehicle control, the probe‐labeled proteins were detected by Western blotting. D) Western blotting assessed the effect of the Cys2768 mutation in UBR5 on PAD4 protein ubiquitination. E) Interaction model between ITA and UBR5 (dashed lines represent hydrogen bonds). F) Interaction model between UBR5 and PAD4 (dashed lines represent hydrogen bonds). n=3 per group for (B‐D). Student's t‐test is used to compare two groups of data affected by a single variable. For the comparison of multiple groups of data, one‐way ANOVA is adopted, and Dunnett's multiple comparisons test is used for post hoc analysis. All data are presented as mean ± standard deviation. Differences were considered statistically significant at *p < 0.05, **p < 0.01, and ***p < 0.001.

ITA is an electrophilic α, β‐unsaturated carboxylic acid that can alkylate cysteine residues in proteins through Michael addition, thereby altering protein activity or function. To determine whether UBR5 is alkylated by the Acod1/ITA axis, we overexpressed Acod1 in PBNs cells and performed proteomic analysis. The results showed that Cys2768 of UBR5 was alkylated by ITA (**Figure** **9**A). Further, we introduced a mutation at the Cys2768 site of UBR5 and used ITalk probes for site‐specific binding validation. Immunoblotting results indicated that ITalk successfully labeled and pulled down wild‐type UBR5 but failed to pull down the C2768S mutant UBR5 (Figure 9B,C), suggesting that Cys2768 is the primary covalent modification site for ITA. We then examined the effect of the Cys2768 mutation on the ubiquitination of PAD4. The results showed that the Cys2768 mutation in UBR5 reversed the enhanced K48‐linked ubiquitination of PAD4 caused by Acod1 overexpression (Figure 9D). Finally, we conducted molecular docking studies of ITA with UBR5 and UBR5 with PAD4 using Vina 1.1.2 software and created binding models for ITA with UBR5 and UBR5 with PAD4 (Figure 9E,F). In summary, Acod1/ITA enhances UBR5 enzyme activity by alkylating the Cys2768 site, which in turn promotes K48‐linked ubiquitination and degradation of PAD4, thereby regulating NETosis and providing protective effects in sepsis.

## 4 Discussion

Sepsis is a complex clinical syndrome triggered by infection and has long posed a significant challenge in global healthcare.^[^ ^28^ ^]^ Its pathophysiological mechanisms involve abnormal activation of the immune system, leading to systemic inflammatory responses and multi‐organ dysfunction.^[^ ^29^ ^]^ Despite some progress in the diagnosis and treatment of sepsis, effective therapeutic options remain limited, especially in early detection and intervention. Therefore, gaining a deeper understanding of the molecular mechanisms of sepsis and identifying new therapeutic targets are crucial for improving patient outcomes.

In this context, our study focused on the role of neutrophils in sepsis, particularly the formation and regulatory mechanisms of neutrophil extracellular traps (NETs). Neutrophils, as key cells in the body's defense system, have their activation and functional abnormalities closely linked to the severity of sepsis.^[^ ^30^ ^]^ NETs, as a novel immune defense mechanism, have attracted considerable attention due to their dual role in sepsis.^[^ ^31^ ^]^ On one hand, NETs can capture and kill pathogens; on the other hand, excessive NETs may cause tissue damage and immune dysfunction.^[^ ^32^ ^]^ Thus, exploring the regulatory mechanisms of NETs holds important clinical significance for the treatment of sepsis.

Our study first revealed the critical role of the Acod1/ITA axis in the immune response of neutrophils in sepsis. By analyzing the peripheral blood PBNs of clinical sepsis patients and sepsis mouse models, we found that the expression level of the Acod1 gene was significantly correlated with the formation of NETs. Acod1 gene knockout mice exhibited more severe inflammatory responses and organ damage in the sepsis model, while Acod1 overexpression alleviated these pathological changes. The main pathways through which Acod1 regulates immunity include the itaconate pathway and the non‐itaconate pathway.^[^ ^33^ ^]^ We mutated the active sites of Acod1 responsible for itaconate production, and combined ELISA and immunofluorescence results showed that, compared with the control group transfected with empty vectors, cells transfected with mutant plasmids (H159Q, L272Q, and H277Y) that do not produce itaconate did not show significant changes in NETs levels. Conversely, cells transfected with WT Acod1 or the N152S plasmid overexpressing itaconate showed significantly reduced NETs levels. This indicated that Acod1 regulates NET production via the itaconate pathway.

Further research revealed that the Acod1/ITA axis regulates the protein level of PAD4, a pivotal protein in NET formation, through the ubiquitin‐proteasome pathway. Protein ubiquitination/deubiquitination modifications play an important role in innate immunity.^[^ ^34^ ^]^ Ubiquitin (Ub), a small molecule protein composed of 76 amino acids, forms various ubiquitin chains depending on its linkage site or polymerization method.^[^ ^35^ ^]^ Among these, K48‐linked ubiquitin chains mainly mediate protein degradation.^[^ ^36^ ^]^ UBR5 (ubiquitin protein ligase E3 component n‐recognin 5) is an E3 ubiquitin ligase containing a HECT domain, capable of mediating K48 and K63‐linked ubiquitination, which is essential for mammalian embryonic development and plays an important role in anti‐inflammatory immune responses.^[^ ^37^ ^]^ Our study found that PAD4 interacts with UBR5 in neutrophils, and UBR5 primarily interacts with the C‐terminal (aa523‐663) domain of PAD4. Therefore, the Acod1/ITA axis regulates PAD4 by enhancing the K48‐linked ubiquitination level and degradation of PAD4 through UBR5.

**Figure 10:**
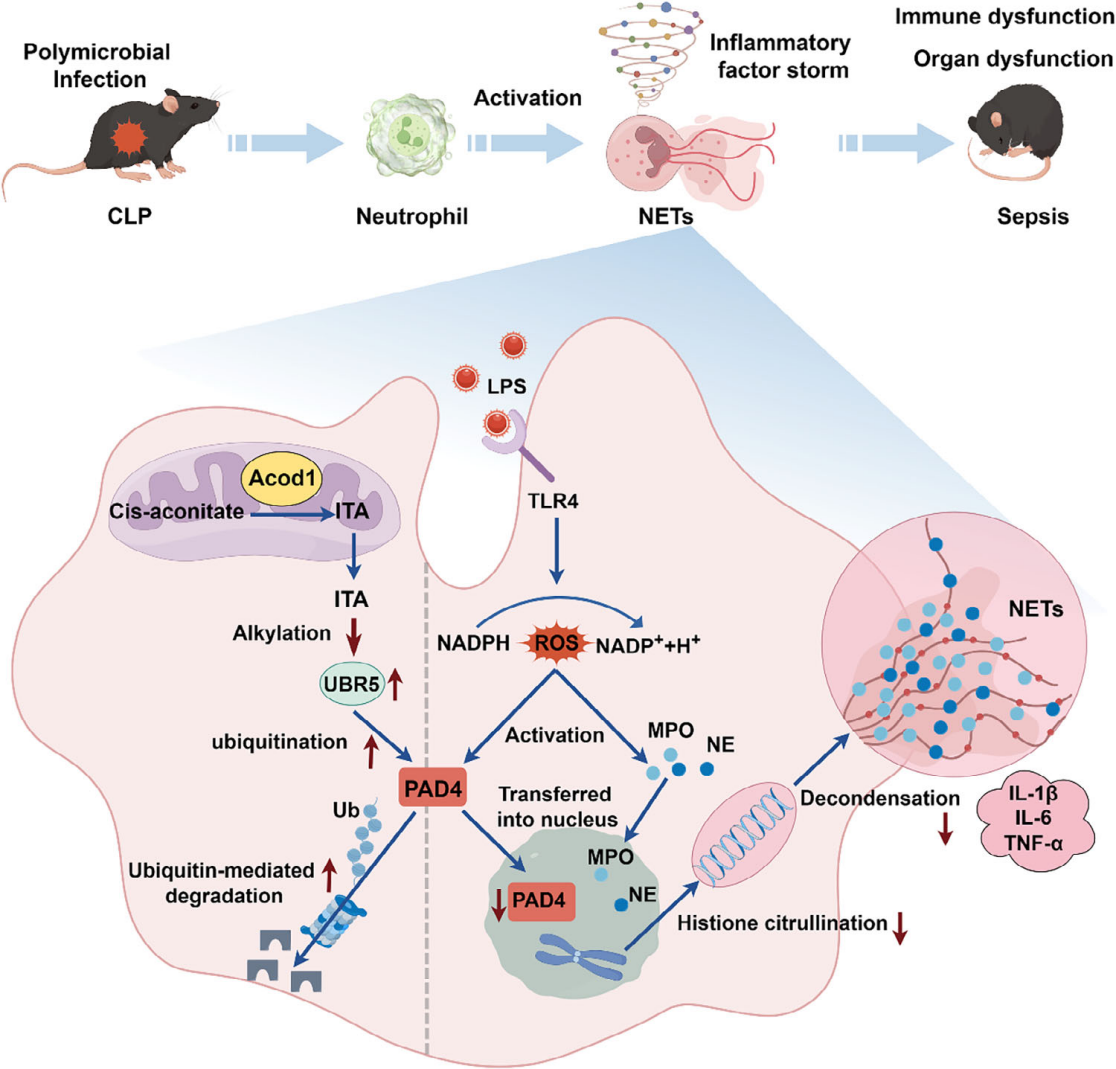
Acod1/ITA exerts protective effects in sepsis by inhibiting NETosis through promoting PAD4 degradation. The Acod1/ITA axis enhances the enzymatic activity of the E3 ubiquitin ligase UBR5 by alkylating the Cys2768 site, thereby facilitating the K48‐linked polyubiquitination and degradation of the PAD4 protein, ultimately modulating NETosis and alleviating inflammatory responses and organ damage in sepsis mice.

One of the key mechanisms by which the Acod1/ITA axis regulates immune responses is the alkylation of target proteins.^[^ ^14^ ^]^ ITA is an electrophilic α, β‐unsaturated carboxylic acid that can alkylate cysteine residues in proteins through Michael addition reactions, forming 2, 3‐dicarboxypropyl adducts, leading to changes in protein function and enzyme activity.^[^ ^38^ ^]^ We hypothesized that the Acod1/ITA axis may enhance the E3 ubiquitin ligase activity of UBR5 by alkylating it, thereby promoting the ubiquitination of PAD4. Proteomics results showed that after overexpression of Acod1 in neutrophils, the Cys2768 site of UBR5 was detected to be alkylated. When the Cys2768 site of UBR5 was mutated, the regulatory effect of Acod1/ITA on PAD4 almost disappeared. In summary, we concluded that the Acod1/ITA axis enhances the enzymatic activity of the E3 ubiquitin ligase UBR5 by alkylating the Cys2768 site, thereby promoting the K48‐linked polyubiquitination and degradation of PAD4 protein, ultimately regulating NETosis and reducing inflammation and organ damage in sepsis mice (**Figure** **10**).

Our study provided new molecular‐level insights into the pathophysiological mechanisms of sepsis and proposed potential therapeutic targets for future interventions. By regulating the Acod1/ITA axis or the downstream UBR5‐PAD4 signaling pathway, and by using 4‐OI with good cell membrane permeability, new therapeutic strategies can be developed, which can effectively reduce the inflammatory burden of sepsis patients and improve their prognosis. These methods not only overcome the limitations of direct application of itaconate (such as poor cell permeability, etc.), but also provide more feasible and advantageous options for clinical treatment. Moreover, our study emphasized the importance of metabolic pathways in regulating immune responses, offering new research directions for exploring immune metabolism in sepsis. However, our study also had certain limitations. First, the study was primarily based on animal models and in vitro cell experiments, and its applicability to human sepsis patients required further clinical validation. Second, although we elucidated the role of the Acod1/ITA axis in regulating NETs formation, its impact on other immune cells and inflammatory diseases remained unclear and needed further investigation. Additionally, our study mainly focused on the direct effects of the Acod1/ITA axis on NETs formation, and the effects on other inflammatory mediators and processes required further exploration.

In conclusion, our study offered a new perspective on the molecular mechanisms of sepsis, particularly in the immune response of neutrophils and NETs regulation. These findings were not only scientifically significant but also provided potential targets for developing new therapeutic strategies. Future research would focus on validating these findings in human sepsis patients and exploring their potential applications in other inflammatory diseases. We hoped that these studies would provide new strategies for the treatment of sepsis, thereby improving patient outcomes and quality of life.

## 5 Conclusion

This study identified Acod1 as a potential key metabolic regulator in sepsis, with its product ITA significantly modulating the formation of neutrophil extracellular traps (NETs). The results indicated that the Acod1/ITA signaling pathway played a crucial role in alleviating sepsis‐related inflammation and organ damage. We elucidated a mechanism where “Acod1/ITA promoted PAD4 ubiquitination and degradation through alkylation modification of UBR5, thereby regulating NETosis.” This finding provided a new direction for therapeutic intervention. The protective effects observed in both in vivo and in vitro sepsis models highlighted the potential of Acod1/ITA as a target for sepsis treatment. Further exploration of this pathway's clinical significance was warranted. This study, integrating perspectives from metabolism and immunology, contributes to a deeper understanding of the pathophysiology of sepsis and laid the foundation for developing new therapeutic strategies.

## Conflict of Interest

The authors declare no conflicts of interest.

## Author Contributions

H.L., G.J., and S.W. contributed equally to this work. H.L., G.J., and S.W.: Designed experiments, wrote the manuscript, and developed the experiments. M.Y., Y.D., and X.C.: Helped to perform the experiments. H.Z., H.G., and J.Z.: Collected the specimens and analyzed the data. X.W. and X.S.: Supervision, Funding acquisition. All the authors have read and approved the final version of the manuscript.

## Supporting information



Supporting Information

## Data Availability

The data that support the findings of this study are available from the corresponding author upon reasonable request.
