# Supplementary material for: Acod1 Promotes PAD4 Ubiquitination via UBR5 Alkylation to Modulate NETosis and Exert Protective Effects in Sepsis
Source: Adv Sci (Weinh). 2025 Jun 30;12(36):e11652. doi: 10.1002/advs.202411652 (PMC12463110; doi:10.1002/advs.202411652)
Supplement: Supplementary file 1 — Supporting Information [file ADVS-12-e11652-s001.docx]

**Acod1 Promotes PAD4 Ubiquitination via UBR5 Alkylation to Modulate NETosis and Exert Protective Effects in Sepsis**

**Huifan Liu ^1#^, Guoqing Jing ^1#^, Shujuan Wu ^3#^, Min Yuan ^2^, Yingyue Dong ^1^, Xue Chen ^2^, Huimin Zhou ^1^, Hailong Gong ^1^, Jing Zuo ^1^, Xiaojing Wu ^2^*, Xuemin Song ^1^***

^1^ Department of Anesthesiology, Research Centre of Anesthesiology and Critical Care Medicine, Zhongnan Hospital of Wuhan University, Wuhan, Hubei, China

^2^ Department of Anesthesiology, Renmin Hospital of Wuhan University, Wuhan, Hubei, 430060, China

^3^ Department of Respiratory and Critical Care Medicine, Renmin Hospital of Wuhan University, Wuhan 430060, China

**^#^** These authors contributed equally to this work

***Correspondence:**

Xuemin Song: Department of Anesthesiology, Research Centre of Anesthesiology and Critical Care Medicine, Zhongnan Hospital of Wuhan University, Wuhan 430071, China.

E-mail: ZN000474@whu.edu.cn

ORCID ID: 0009-0009-5788-7857

Xiaojing Wu: Department of Anesthesiology, Renmin Hospital of Wuhan University, Wuhan, Hubei, 430060, China.

Email: RM000851@whu.edu.cn


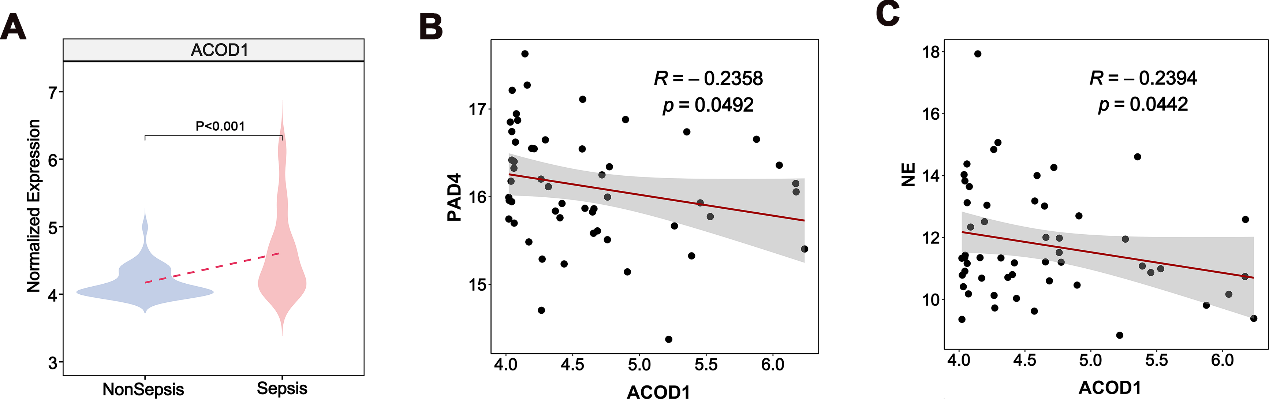


**Figure S1.** (A) Partial RNA sequencing data from the GEO dataset (GSE154918) showed the expression differences of the Acod1 gene between healthy volunteers and clinical sepsis patients; (B) The correlation between Acod1 gene expression levels and PAD4 gene expression levels; (C) The correlation between Acod1 gene expression levels and NE gene expression levels. n=20 per group (A-C). Student's t-test is used to compare two groups of data affected by a single variable. Data are displayed as mean ± standard deviation. Differences were considered statistically significant at **p* < 0.05, ***p* < 0.01, and ****p* < 0.001.


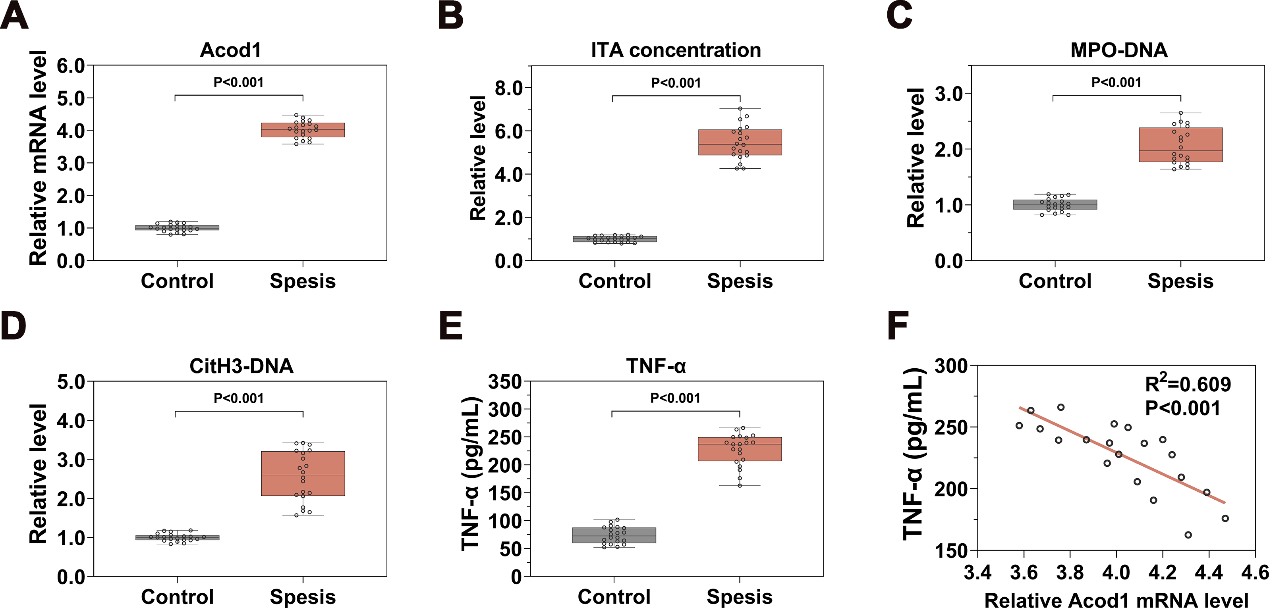


**Figure S2.** (A-B) The mRNA level of Acod1 and the level of itaconate (ITA) in peripheral blood neutrophils of healthy volunteers and sepsis patients; (C-E) The levels of MPO-DNA, CitH3-DNA and the concentration of TNF-α in peripheral blood of healthy volunteers and sepsis patients; (F) Pearson correlation analysis between the concentration of TNF-α and the mRNA level of Acod1 in peripheral blood of sepsis patients. Student's t-test is used to compare two groups of data affected by a single variable. For the comparison of multiple groups of data, one-way ANOVA is adopted, and Dunnett's multiple comparisons test is used for post hoc analysis. All data are presented as mean ± standard deviation. Differences were considered statistically significant at **p* < 0.05, ***p* < 0.01, and ****p* < 0.001.


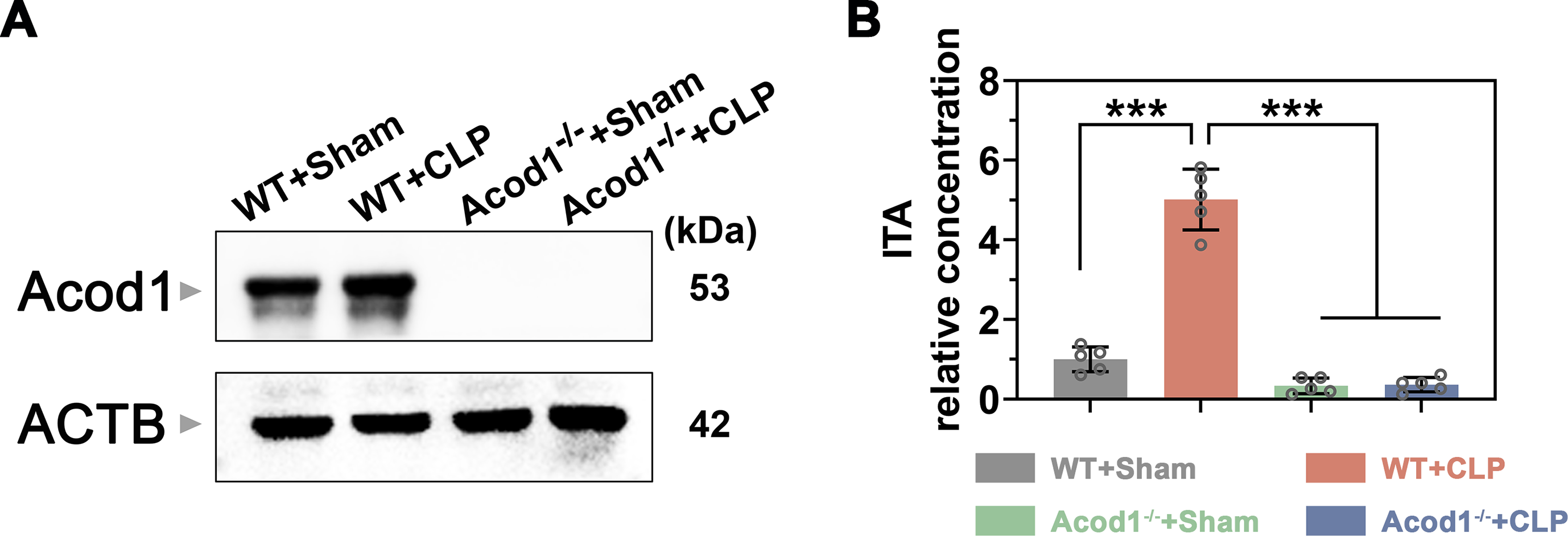


**Figure S3.** (A) Western blotting analysis confirmed the successful knockout of Acod1 in PBNs from Acod1^-/-^ mice. (B) High-performance liquid chromatography analysis measured ITA levels in PBNs from mice. n=3 per group for A, n=5 per group for B. Student's t-test is used to compare two groups of data affected by a single variable. For the comparison of multiple groups of data, one-way ANOVA is adopted, and Dunnett's multiple comparisons test is used for post hoc analysis. All data are presented as mean ± standard deviation. Differences were considered statistically significant at **p* < 0.05, ***p* < 0.01, and ****p* < 0.001.


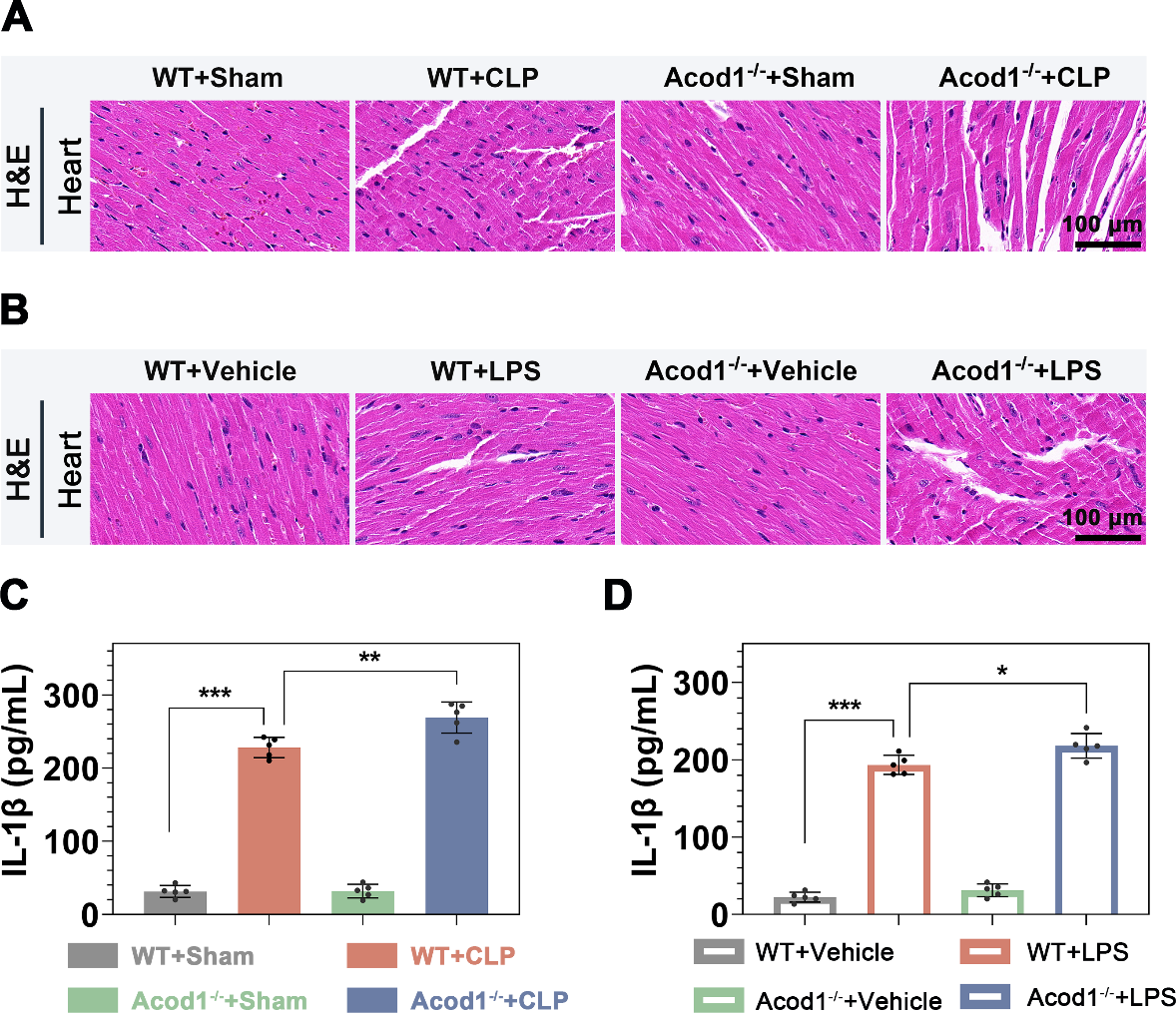


**Figure S4.** (A) After CLP surgery, the degree of injury to the mouse heart tissue was evaluated by HE staining (scale bar = 100 μm); (B) After intraperitoneal injection of LPS, the pathological damage of the mouse heart tissue was evaluated by HE staining (scale bar = 100 μm). (C) After CLP modeling, ELISA was used to detect the level of the pro-inflammatory cytokine IL-1β in the peripheral blood of mice; (D) After LPS injection modeling, ELISA was used to detect the level of the pro-inflammatory cytokine IL-1β in the peripheral blood of mice. n=5 per group. Student's t-test is used to compare two groups of data affected by a single variable. For the comparison of multiple groups of data, one-way ANOVA is adopted, and Dunnett's multiple comparisons test is used for post hoc analysis. Differences were considered statistically significant at **p* < 0.05, ***p* < 0.01, and ****p* < 0.001.


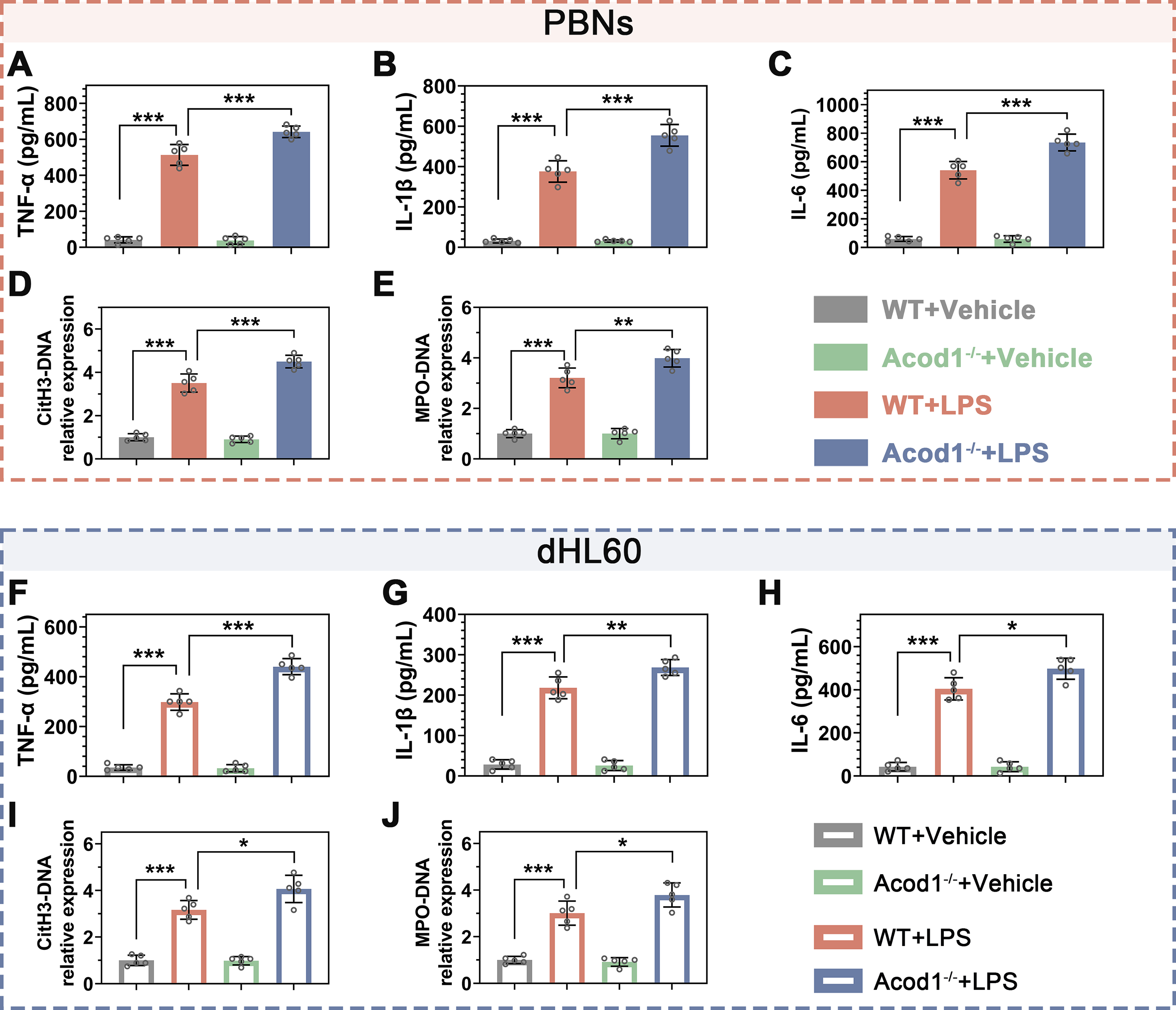


**Figure S5.** (A-C) After collecting the culture supernatant of mouse PBNs, the levels of inflammatory factors TNF-α, IL-1β, and IL-6 were measured using the ELISA method. (D-E) Additionally, the relative levels of NETs markers CitH3-DNA complexes and MPO-DNA complexes were assessed . For the dHL60 cell culture supernatant, the levels of TNF-α, IL-1β, and IL-6 were also measured using the ELISA method (F-H), and the relative levels of CitH3-DNA complexes and MPO-DNA complexes were determined (I-J). n=5 per group. Differences were considered statistically significant at **p* < 0.05, ***p* < 0.01, and ****p* < 0.001.


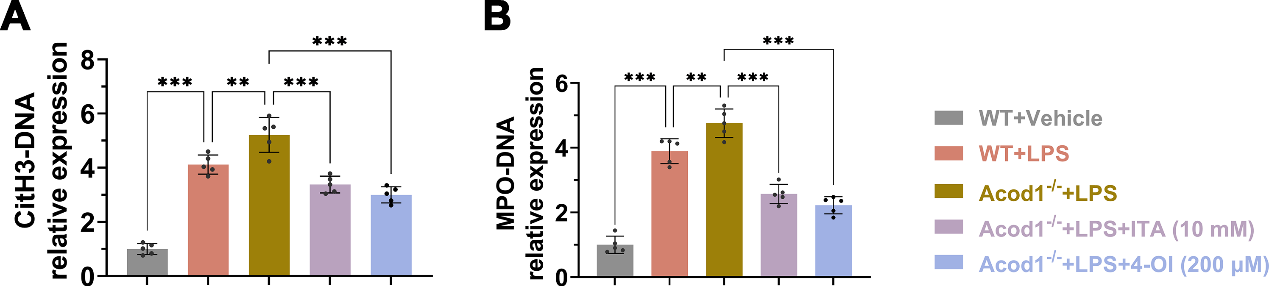


**Figure S6.** (A-B) The concentrations of NETs markers CitH3-DNA and MPO-DNA complexes in the cell supernatants were measured by ELISA. n = 5 per group. Differences were considered statistically significant at **p* < 0.05, ***p* < 0.01, and ****p* < 0.001.


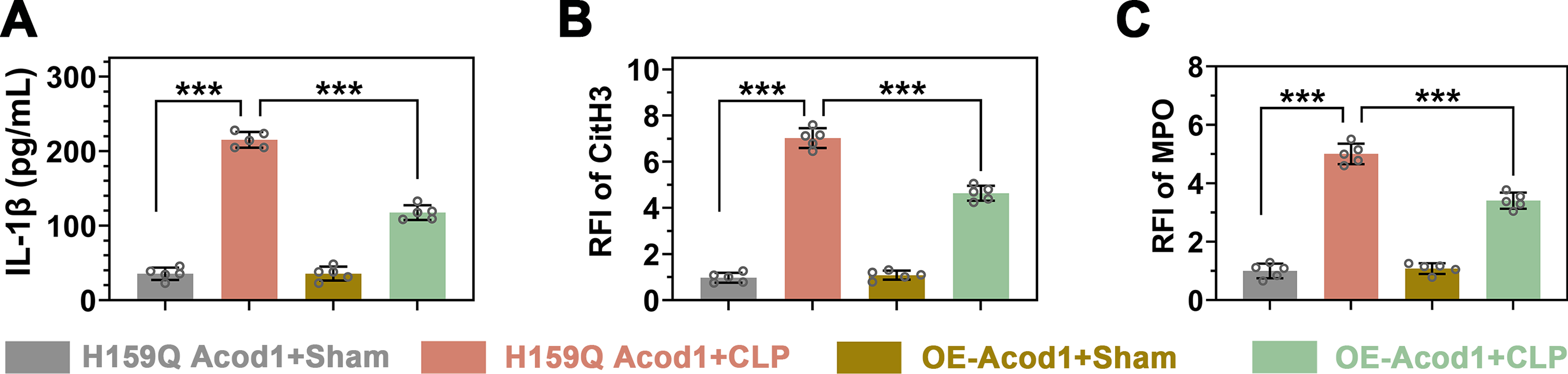


**Figure S7.** After CLP modeling, (A) the level of the pro-inflammatory cytokine IL-1β in the peripheral blood of mice was measured using ELISA; (B-C) quantitative analysis of the immunofluorescence images of MPO and CitH3 in mouse lung tissue was conducted (Figure 5K). n = 5 per group. Differences were considered statistically significant at **p* < 0.05, ***p* < 0.01, and ****p* < 0.001.


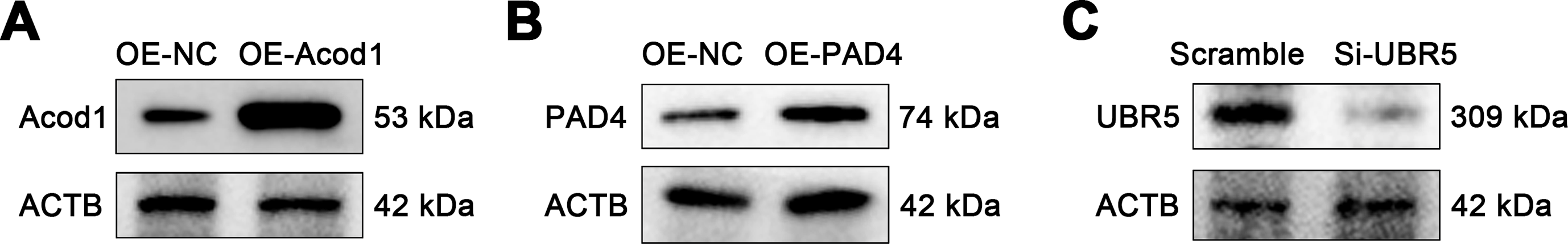


**Figure S8.** Validation of Western blotting results: (A) Overexpression of Acod1, (B) Overexpression of PAD4, and (C) Knockdown of UBR5. n=3 per group.


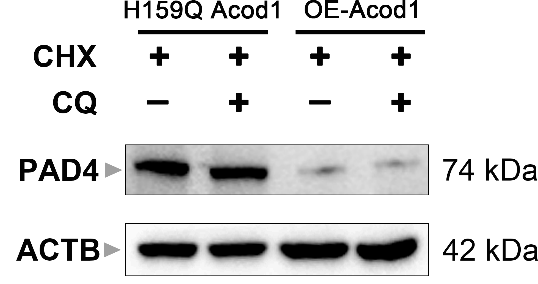


**Figure S9.** PBNs cells were treated with CHX and stimulated with chloroquine (CQ) for 9 h. The PAD4 protein was determined by Western blotting. n=3 per group.

| **Clinical data** | **Control group (*n* = 20)** | **Sepsis group (*n* = 20)** | ***P*** |
| --- | --- | --- | --- |
| Age (year, mean ± SD) | 47.75 ± 20.50 | 51.25 ± 14.00 | >0.05 |
| Gender (male/female) | 10/10 | 12/8 | > 0.05 |
| WBC (× 10^9^/L, mean ± SD) | 4.75 ± 1.25 | 13.55 ± 6.95 | < 0.05 |
| CRP (mg/dl, mean ± SD) | 1.10 ± 0.55 | 10.50 ± 6.35 | < 0.05 |
| SOFA score (mean ± SD) | 0 | 7.70 ± 2.60 | < 0.001 |
| APACHE Ⅱ score (mean ± SD) | 0 | 18.55±4.27 | < 0.001 |

**Table S1. Baseline characteristics of the study population**

**Table S2. Primary antibodies used in this study**

| Target | Host | Dilution used | Manufacturer | Catalog Number |
| --- | --- | --- | --- | --- |
| ACTB | Mouse | 1:5000 | Abmart | T40104 |
| CitH3 | Rabbit | 1:1000 | Abcam | ab5103 |
| MPO | Rabbit | 1:1000 | Abcam | ab272101 |
| NE | Rabbit | 1:1000 | Abcam | ab314916 |
| Acod1 | Rabbit | 1:1000 | Proteintech | 28436-1-AP |
| UBR5 | Mouse | 1:1000 | Proteintech | 66937-1-Ig |
| Flag | Rabbit | 1:5000 | Proteintech | 20543-1-AP |
| Myc | Mouse | 1:5000 | Proteintech | 60003-2-Ig |

**Table S3. Primer sequences used in qRT-PCR analysis.**

| **Genes** | **Primers (F, forward; R, reverse; 5’-3’)** |
| --- | --- |
| Mouse-ACTB | F: TGACGTTGACATCCGTAAAG |
|  | R: GAGGAGCAATGATCTTGATCT |
| Mouse-PAD4 | F: GACCACAACAGTTCTCGTATTGC |
|  | R: CGGGTTAGACTTGTCCAGCAG |
| Mouse-MPO | F: TGCTTCCTGGCAGGGGA |
|  | R: CCACCTAG GGTTCAGGCTCT |
| Mouse-NE | F: GTGGCGAATGTAAACGTC |
|  | R: CCGTTGAGCTGGAGAATC |
